# Consumer Adoption of Personal Health Record Systems: A Self-Determination Theory Perspective

**DOI:** 10.2196/jmir.7721

**Published:** 2017-07-27

**Authors:** Vahid Assadi, Khaled Hassanein

**Affiliations:** ^1^ McMaster Digital Transformation Research Centre DeGroote School of Business McMaster University Hamilton, ON Canada

**Keywords:** health records, personal, health care information systems, online systems, technology, intention, physician patient relationships, personal autonomy, psychological theory, social theory, behavior

## Abstract

**Background:**

Personal Health Records (PHR) systems provide individuals with access and control over their health information and consequently can support individuals in becoming active participants, rather than passive recipients, in their own care process. In spite of numerous benefits suggested for consumers’ utilizing PHR systems, research has shown that such systems are not yet widely adopted or well known to consumers. Bearing in mind the potential benefits of PHRs to consumers and their potential interest in these systems—and that similar to any other type of information system, adoption is a prerequisite for realizing the potential benefits of PHR systems—research is needed to understand how to enhance the adoption rates for PHR systems.

**Objective:**

This research seeks to understand how individuals’ intentions to adopt PHR systems are affected by their self-determination in managing their own health—the extent of their ability to take an active role in managing their own health. As such, this research aims to develop and empirically validate a theoretical model that explains PHR systems adoption by the general public through the integration of theories from the information systems and psychology literatures.

**Methods:**

This research employs a cross-sectional survey method targeted at the Canadian general public without any prior experience in using PHR systems. A partial least squares approach to structural equation modeling was used to validate the proposed research model of this study (N=159).

**Results:**

Individuals with higher levels of ability to manage their own health (self-determination) are more likely to adopt PHR systems since they have more positive perceptions regarding the use of such systems. Further, such self-determination is fueled by autonomy support from consumers’ physicians as well as the consumers’ personality trait of autonomy orientation.

**Conclusions:**

This study advances our theoretical understanding of PHR systems adoption. It also contributes to practice by providing insightful implications for designing, promoting, and facilitating the use of PHR systems among consumers.

## Introduction

### Background

A personal health record (PHR) system is an information system that comprises data as well as supporting tools and functionalities related to an individual’s health. The most cited definition of a PHR system [1,2] was put forth by the Markle Foundation [3] as: “An electronic application through which individuals can access, manage, and share their health information, and that of others for whom they are authorized, in a private, secure, and confidential environment.”

Personal health records are created, owned, updated, and controlled by individual consumers or others authorized by them. Ideally, they contain a summary of the consumer’s lifelong health information such as their history of previously undertaken health procedures, major illnesses, allergies, home monitoring data (eg, blood pressure), family history, immunizations, medications, and laboratory test results [4]. Such access to health records can be leveraged with the support of tools and functionalities for the purpose of better managing one’s health [1,5,6]. Examples of such functionalities include allowing the consumer to communicate electronically with clinicians [1] and to share records with clinicians [7]. The PHR definition in this paper is consistent with that in International Organization for Standardization Technical Report 14292 [8]. Throughout this paper, the words “consumer,” “individual,” and “patient” are used interchangeably since PHR system consumers are not necessarily dealing with immediate medical concerns and can be ill or healthy.

When successfully implemented and used, PHR systems have the potential to facilitate a transformative advancement in health care delivery and management. Such advancements are likely to be in the form of “improved interactions between patients and care providers,” increased “opportunities to realize innovation in care management,” “a shift in the locus of control of health information” to a more shared control between patients and care providers, and improved “efficiency in care” [5,6].

In spite of numerous benefits suggested for consumers’ utilizing PHR systems [9-14], research has shown that such systems are not yet widely adopted or well known to consumers [9,10,15-23]. Bearing in mind the potential benefits of PHRs to consumers and their potential interest in these systems [11,24,25]—and that similar to any other type of information system (IS), adoption is a prerequisite for realizing the potential benefits of PHR systems [26]—research is needed to understand how to enhance adoption rates for PHR systems.

By providing individuals with access and control over their health information, PHR systems can support individuals in becoming active participants, rather than passive recipients, in their own health care process [1,2,27-29]. However, for such systems to provide the right support for their user, the user must disburse an “ongoing” effort to keep their account up to date. Such an ongoing effort reduces the amounts of (and likelihood of) outdated, inaccurate, or incomplete information in the record, which could result in the wrong health care decisions being made [1]. PHR systems are examples of an emerging class of information systems that, through providing access and control to useful information with an associated need for ongoing maintenance (eg, updating one’s health record regularly) which entails a significant effort, support individuals in taking a more active role in the context for which the information system is designed [30,31]. Examples of such systems in other contexts include social networking systems (social context) and personal finance management systems (financial context). We argue that users of such systems must accept and be able to take a more active role in managing the behavior supported by the information systems (eg, managing their own health). The users must espouse the appropriate personal traits and be supported by the right environmental factors to facilitate their taking on such an active role. This provides them with the appropriate level of motivation to use such systems in spite of the required ongoing maintenance effort [31]. From this perspective, understanding the adoption of such systems warrants augmenting existing information systems and PHR adoption models.

Hence, this research seeks to understand how the extent of individuals’ ability to take a more active role in managing their health could affect their intentions to adopt electronic PHR systems. We further seek to understand some of the important personal and environmental antecedents that support the individual in accepting and practicing such an active role in their own health management. This is accomplished by proposing a PHR systems adoption model through the integration of theories from the information systems and psychology literatures. This model is then validated through an empirical study involving a stratified sample of 159 Canadians, leading to important results with implications for theory and practice.

### Objectives

Several studies have investigated the factors responsible for the lack of PHR adoption (eg, [1,16,32-41]). Of particular interest to our study, behavioral and environmental factors are suggested to impact PHR system adoption [1]. It is widely believed that proper use of PHR systems would support a change in the role of consumers from passive recipients of treatment to active partners (with health care providers) in their health management process (eg, [1,2,27-29]). Such partnership includes, for example, consumers’ seeking health information [42], managing their own health and wellness [1,5,19,43], and becoming more involved in their health care decision making [19]. As such, a PHR system can be more useful for the individual owner only if they understand and accept a more active role as well as new responsibilities related to their own health care [1]{Formatting Citation}. The influence of consumers’ ability (readiness) to take such an active role in their adoption of PHR systems is not examined in the literature. As such, this study draws on information systems and psychology literature to understand how consumers’ readiness to take a more active role in their health and wellness management could influence their adoption of PHR systems. The existing IS literature also calls for research that helps us understand what would give rise to “effective” IS use, rather than just IS use [44], which is in line with the arguments made above for observing the conditions (ie, considering the aforementioned role change) for effective PHR usage.

To understand how to enhance PHR adoption rates, we integrated mainstream IS adoption models with Self-Determination Theory (SDT), which is a theory of motivation from the field of psychology. SDT sheds light on the mechanisms through which individuals become able and motivated to take active (rather than passive) roles when engaging in different types of behaviors including individual health care [45]. As such, the justification for augmenting IS adoption models with SDT in this context is twofold. First, motivation is generally an important consideration in IS adoption [46,47]. More specifically, lack of proper motivation has been identified as a major inhibitor for adoption of PHR systems [1]. Second and as explained above, for PHR systems to be useful requires consumers to understand and accept (ie, being able and ready for) a change in their roles in their own health management, from passive to active [1]. As such, the main objectives of this research are to (1) develop and empirically validate a model to understand how individuals’ perceptions of the extent to which they feel able in managing their own health (self-determination) would influence their intentions to adopt PHR systems, and (2) assess the impacts of the environmental factor of physician autonomy support and the behavioral factor of individuals’ autonomous causality orientation on their perceptions of being self-determined in their health management behavior.

This paper builds, in part, on existing studies on PHR systems in order to develop and validate an adoption model for PHR systems while maintaining novelty by being the first to observe the following unique set of characteristics: (1) it (the paper) is targeted at the general public and not a specific segment of the population, (2) it focuses on integrated PHR systems—systems that gather and present data from multiple sources (eg, consumer, care provider, health care organizations) into a single view, generally through secure Internet access [2,5,48], (3) it is not disease specific (ie, relates to health and wellness management in general), (4) it is grounded in theory as it integrates mainstream IS adoption models with SDT, and (5) it employs a rigorous hypothetico-deductive method for validation of findings.

Multimedia Appendix 1 provides the positioning of this paper in relation to the existing studies in great detail.

### Theoretical Development

#### Viability of Self-Determination Theory to Help Explain Personal Health Record System Adoption

A PHR system has the potential to empower individual owners to play a more active role in their health management [28,49-52]. Thus, a PHR system can be more useful for the individual if they understand and accept (ie, are able and ready for) this more active role (in their health management) as well as the new responsibilities associated with this active role [1]{Formatting Citation}. From psychology literature, SDT is a theory that is potentially useful for explaining ability and motivation in the context of individual health management. SDT explains the mechanism through which individuals become able and motivated to take more active (rather than passive) roles in engaging in different types of behaviors including individual health care [45]. This theory is also considered to be the guiding principle of patient empowerment [53]. It is also believed that this theory is well suited for understanding the role of information technology in consumer-based health care [49]. Finally, given the frequent calls for theory development in this context (eg, Pingree et al [54]) as well as the need for clarifying the IS “nuances” involved in IS adoption research [55], integrating SDT with mainstream IS adoption models, in order to explain the adoption of PHR systems, is both promising and necessary. As such, this integration serves the overall objective of this research.

SDT represents a broad framework for the understanding of human motivation and personality. SDT begins with the assumption that human beings are active organisms with evolved tendencies toward growing, mastering new skills, applying their talents responsibly, learning, and integrating new experiences into a sense of self. As such, they tend to behave in a “self-determined” way [45]. SDT asserts that the more a behavior is self-determined for an individual, the more the individual will be able and motivated to take an active (rather than passive) role in that behavior. However, such human tendencies and self-determination require ongoing support from the social environment. Without such ongoing support, human spirit can be diminished, and individuals might reject growth and responsibility [45].

**Figure 1:**
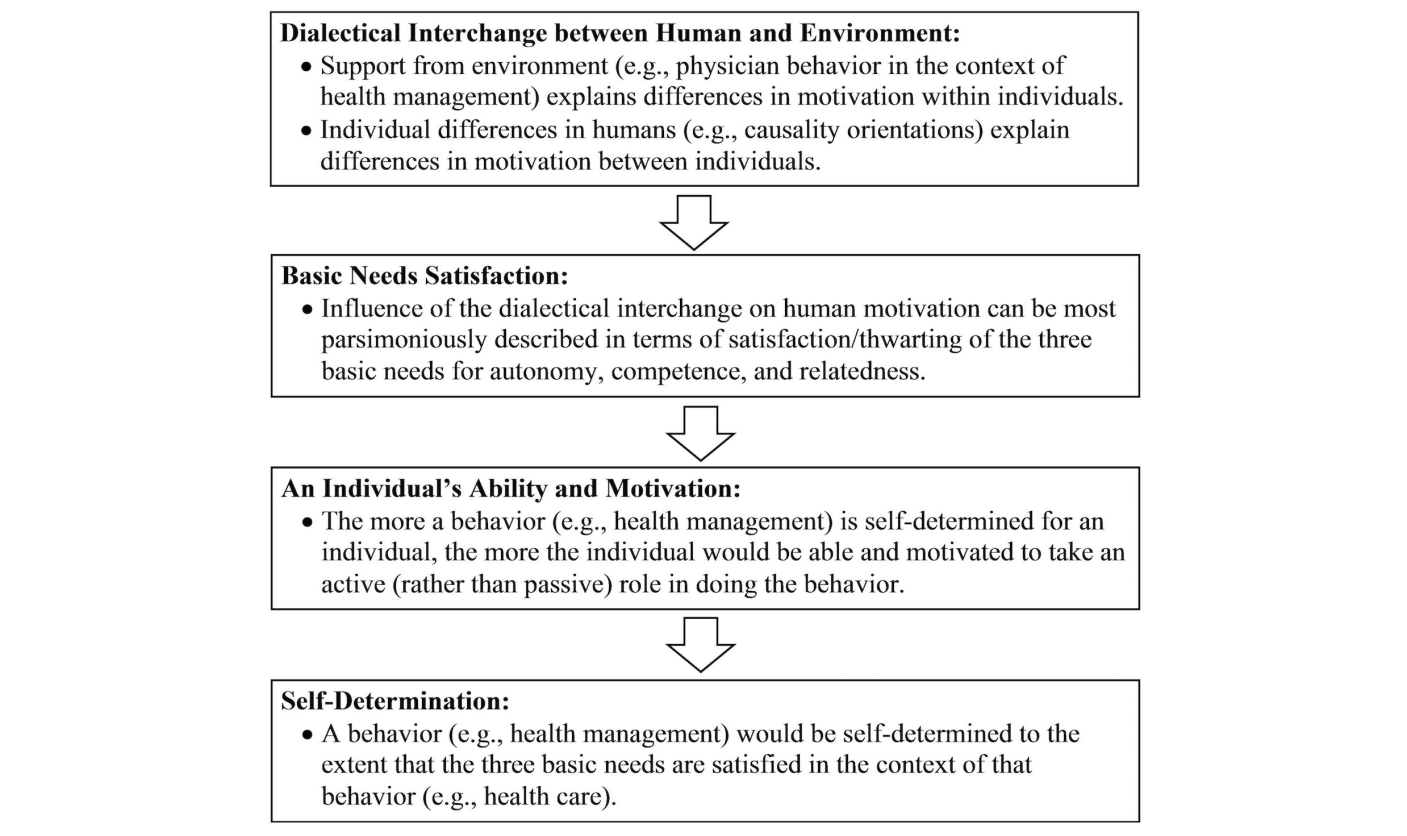
How an individual could take an active (rather than passive) role in their health management, according to SDT.

Research guided by SDT shows the importance of “environmental” (eg, physician behavior) and “consumers’ personality characteristics” in explaining differences in self-determination and motivation, “within” and “between” individuals respectively [45]. According to SDT, such differences can be most parsimoniously described in terms of their effect on the satisfaction/thwarting of three basic psychological needs for autonomy, competence, and relatedness [45,56]. The more the three needs are satisfied for an individual in the context of a given behavior, the more able and ready the individual will feel to engage in the behavior actively (ie, self-determined) [45,56]. The need for autonomy refers to an individual’s desire to self-organize their behavior, when they feel the desire to do so [57,58]. Competence concerns the individual’s belief about their capabilities in performing an action in a social context [59,60]. The need for relatedness refers to the individual’s desire to feel socially connected and supported, especially by important others, such as managers, teachers, or health care providers (Figure 1; adapted from [45,56,61,62]).

Throughout the years, SDT has been successfully applied [45,56,57] in many research domains including work organizations (eg, [63,64]), virtual environments and media (eg, [65-67]), and health care (eg, [61,68-71]).

**Table 1:** Construct definitions.

Construct	Definition
Autonomous Causality Orientation	A person’s tendency toward being autonomous (ie, self-determined) in general, across different domains and times [72].
Physician Autonomy Support	The extent to which physicians obtain and acknowledge patients’ perspectives, support their ideas, offer choices in treatment options, and offer relevant information without trying to pressure them [70].
Basic Needs Satisfaction	A measure of self-determination in a given context assessed through the satisfaction/thwarting of the three basic needs for autonomy, competence, and relatedness [73].
Self-Efficacy	An individual’s belief of having the capability to use computers [74,75].
Complexity	The degree to which a PHR system is perceived as relatively difficult to understand and use [76].
Perceived Usefulness	The extent to which an individual believes that a PHR system can be used advantageously in managing their health [77].
Behavioral Intention	A measure of the strength of an individual’s intention to use a PHR system for managing their health [78].

#### Proposed Theoretical Model and Hypotheses Development

**Figure 2:**
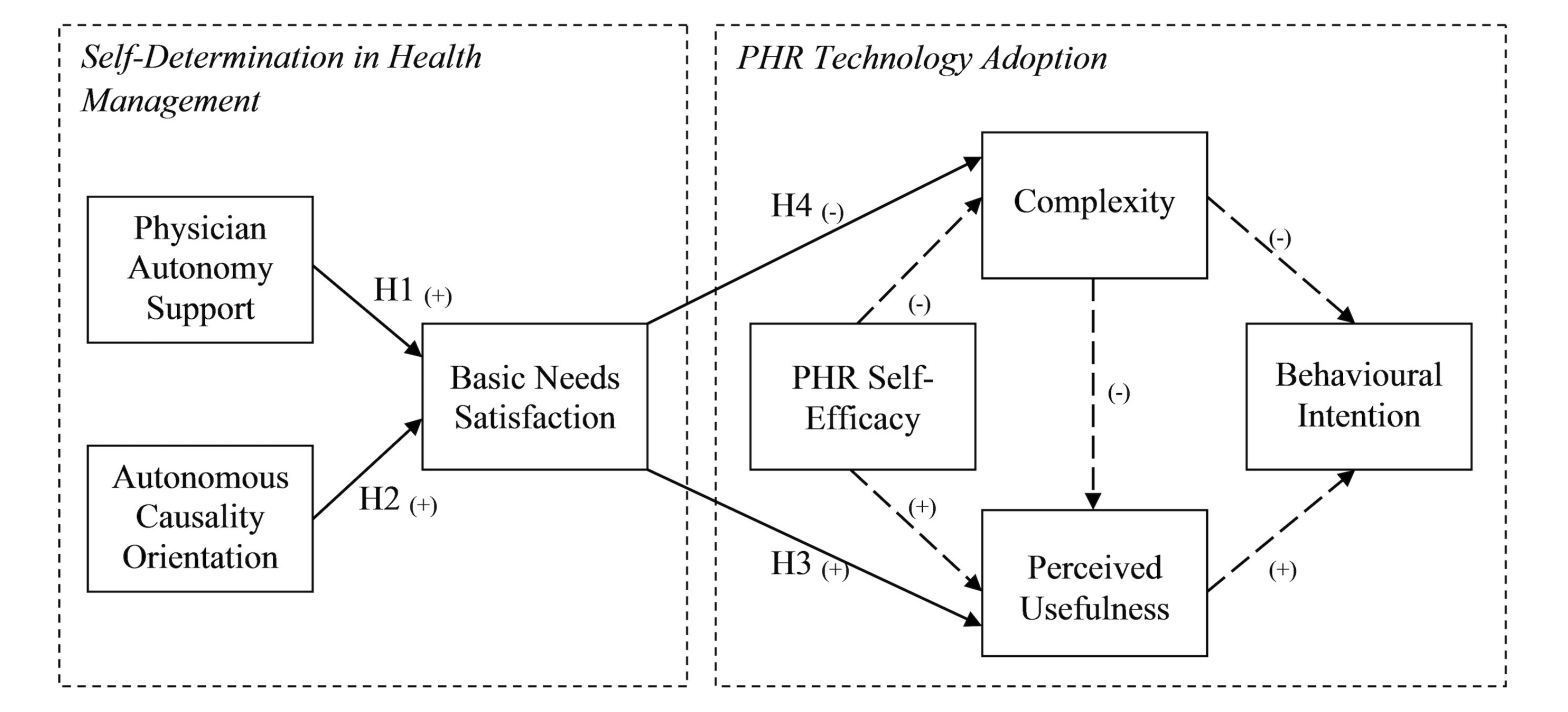
Research model and hypotheses (arrows in bold demonstrate the main focus of this study; dashed lines on the right side of the model are included for statistical testing but are not specifically hypothesized as they have been repeatedly established in IS literature).

The proposed theoretical model of this study (Figure 2) is composed of two main components: PHR Technology Adoption and Self-Determination in Health Management. This section describes the development of each of these components, followed by a description of the link between them.

The definitions of all the constructs in the model are shown in Table 1. In the rest of this section, the rationale for this model is provided and the solid-line hypotheses (Figure 2) are developed with appropriate support.

The PHR Technology Adoption component of the model (Figure 2, right side) was developed based on TAM. Extensive research on IS adoption (eg, [77,79-87]) has demonstrated that TAM [77] represents the most parsimonious essence of IS adoption theories. TAM has consistently been shown to explain IS adoption across various contexts [88] and stages (pre-usage to post-usage) of IS adoption [89]. Accordingly, and given TAM’s centrality in IS adoption research, it is desirable to use for covering the technology adoption side of this research.

TAM holds that an individual’s behavioral intention to use an IS is mainly determined by their beliefs regarding the usefulness (Perceived Usefulness – PU) and ease of use (Perceived Ease of Use – PEOU) associated with using that IS [77]. Prior research in IS and reference disciplines has shown the role of behavioral intention as a strong predictor of actual use (eg, [81,82,90-94]). Therefore, and given the relatively small number current users of PHR systems, behavioral intention to use, rather than actual use, is incorporated as the endogenous variable in the Figure 2 model.

As noted from Figure 2, PEOU was replaced with a similar yet distinct measure of ease of use (ie, complexity), which we deemed more suited for the context of this study. PEOU is defined as “the degree to which a person believes that using a particular system would be free of effort” [77]. PEOU, as well as its most associated measurement scales, relates to the ease (or effort) to “initially” learn how to operate a system. Example items of the measurement scale for the PEOU construct are “learning to operate the system would be easy for me” [77]. Research has shown that PEOU’s effect on adoption ceases to become significant past this initial phase of learning how to operate the system [81,82]. However, proper use of a PHR system requires efforts beyond the “initial” learning effort. A PHR system owner or user must disburse an “ongoing” effort to keep their account up to date. Such an ongoing effort reduces the amounts of (and likelihood of) outdated, inaccurate, or incomplete information in the record, which could result in the wrong health care decisions being made [1]. Therefore, for the context of this study, a construct and associated measurement scale that, in contrast to PEOU, captures such ongoing effort was incorporated.

In a review of technology adoption models/constructs, Venkatesh [80] identified several constructs (including PEOU) as root constructs of effort expectancy. Relative to other identified constructs, “complexity” (CPLX) [76] better encapsulates both the effort required to “initially” learn how to use an IS as well as the “ongoing” effort to keep the system up to date. Example items from the measurement scale for CPLX include “It takes too long to learn how to use the system to make it worth the effort” (ie, effort to initially learn how to use the system), “Using the system involves too much time doing mechanical operations” (eg, data input on an ongoing basis), and “Using the system would take too much time from my normal duties” (ie, ongoing effort beyond the initial learning effort). In light of this argument, CPLX was incorporated in the Figure 2 model to represent perceptions of effort associated with using PHR systems. As such, it is used in the model as a direct negative determinant of both behavioral intention and PU [76,77,82,94,95].

Finally, a construct that has been consistently shown to determine user perceptions of IS, especially in the early stages of adoption, is that of self-efficacy [74,80,82,96]. Computer self-efficacy refers to an individual’s belief of having the capability to use computers [74,75]. This definition can be extended to the belief of having the capability to use an Internet app such as an integrated PHR system (PHR self-efficacy). Since this study aims to understand the pre-usage intentions to use an integrated PHR system, it is important to consider investigating the influence of self-efficacy on adoption. Consequently, PHR self-efficacy is incorporated in Figure 2 as a direct determinant of both PU (positive) and CPLX (negative).

**Figure 3:**
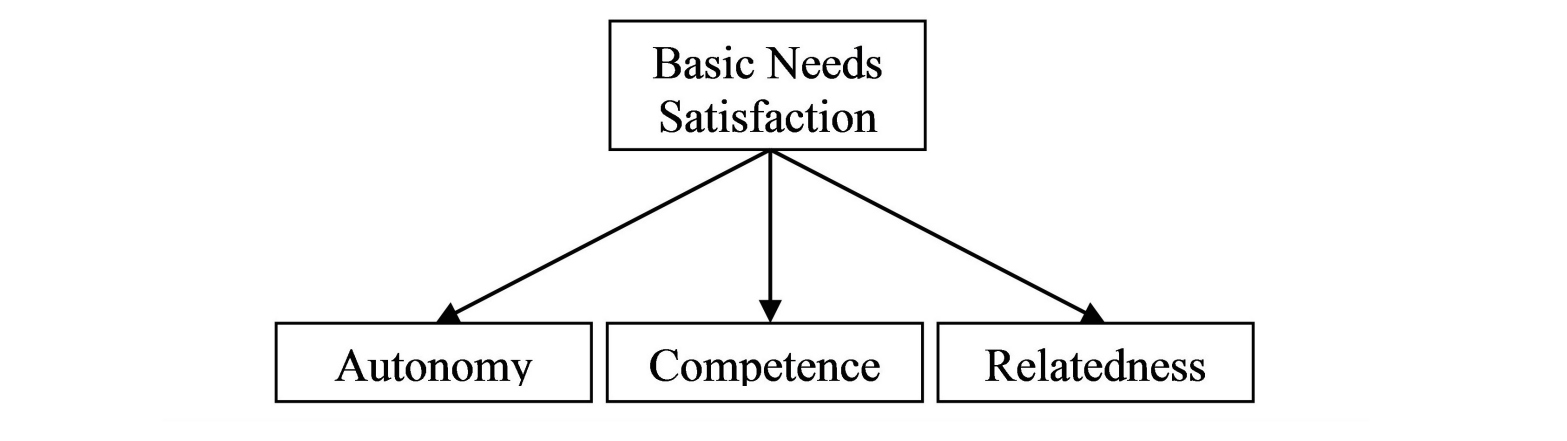
Modeling of basic needs satisfaction as a second-order construct.

The second component of the proposed research model (Figure 2, left side) was developed based on SDT. As explained in the previous section (Figure 1), self-determination in a given context can be most parsimoniously described in terms of the satisfaction/thwarting of the three basic needs for autonomy, competence, and relatedness—Basic Needs Satisfaction (BNS). The authors of SDT mention that satisfaction of the three needs must happen together to have positive effects on self-determination [45,73,97]. As a result, in the current study, BNS was modeled as a second-order construct, following Deci et al [73] (Figure 3).

According to SDT, and as seen in Figure 1, self-determination flourishes in an environment that supports the satisfaction of the three basic needs for autonomy, competence, and relatedness (ie, BNS). SDT asserts specifically that the more autonomy supportive a superordinate is (eg, teacher, manager, physician as elements of social environment surrounding an individual), the more satisfied the three basic needs of a subordinate will be (eg, student, employee, patient) [45]. This influence is demonstrated in several studies in various contexts. Examples include the positive influence of physician autonomy support in the context of diabetes self-management [70,98], physician autonomy support in the context of patient weight loss [70,98,99], supervisor autonomy support in a work organization [73,99-101], and parent autonomy support in promoting children’s prosocial behavior [97]. As a representative of a superordinate’s autonomy orientation in SDT, in a health care context, physician autonomy support has been consistently shown to influence the satisfaction of the three needs in the physician’s patient(s), which makes health management more self-determined for patients [45,102]. In addition, research in the context of PHR systems suggests that for PHR systems to be useful, health care providers in general, and physicians in particular, must support the changing roles of their patients by encouraging them to maintain their records and by appropriately trusting information provided by patients [1]. Finally, research shows that providers’ use of PHR messaging would encourage the individuals to do so [103,104]. Based on this discussion, the following is hypothesized:

H1: A higher level of perceived physician autonomy support positively influences an individual’s level of BNS in the context of health management.

Several studies that have employed SDT in different contexts have shown a positive association between autonomous causality orientation and BNS. Example contexts include weight loss [71], work organization [63], and promoting prosocial behavior in children [97]. Thus, the following is hypothesized:

H2: A higher level of an individual’s autonomous causality orientation is positively associated with their level of BNS in the context of health management.

Hypotheses H3 and H4 in the proposed research model in Figure 2 pertain to investigating possible associations between BNS in the context of health management and an individual’s beliefs regarding the use of PHR systems to help manage one’s health. As mentioned earlier, for a PHR system to be useful, the individual owner should understand and accept (ie, being able and ready for) a more active role in their health management. Such an active role requires motivation [1]. SDT formulates this ability in the form of the satisfaction of the three basic needs for autonomy, competence, and relatedness [45]. Higher levels of BNS in the context of health management would result in health management behaviors to become more self-determined (for an individual) [45]. We argue that an individual who is more self-determined in their health management would have more positive beliefs regarding the use of PHR systems. This argument is made based on the following logical justification: (1) PHR systems are suggested to support consumers’ self-determination in managing their health (eg, [24,105-110]), (2) consumers desire to become empowered and self-determined in managing their own health (eg, [24,94,111,112]), (3) higher levels of BNS make an individual better able to take a more active role in their health care [45], and (4) beliefs of usefulness and effort associated with using an IS are considered motivational factors to make use of that IS [113,114].

Based on the four justifications above, it is reasonable to hypothesize that consumers with higher levels of self-determination (associated with higher BNS) in managing their health would have more positive beliefs (eg, higher PU and lower CPLX) regarding the use of a technology that supports their reaching their goal.

In a survey of consumers’ perceptions regarding the use of PHR systems, PHR system functionalities that were rated highest (in terms of usefulness) among survey respondents were the ones that aligned with the satisfaction of SDT’s three basic needs (for autonomy, competence, and relatedness) [49]. In addition, higher self-determination means higher ability and motivation to take an active role in health management [45]. They are therefore likely to perceive less effort and complexity in using a system that is designed to help them take more responsibility in their health management. Based on SDT, more self-determined individuals are likely to have more intrinsic/internalized motivation to manage their health [45,115]. Thus, they are likely to perceive less effort [116] associated with using a tool that is designed to support self-determination in health management [24,28,105]. We thus hypothesize the following:

H3: A higher level of BNS in the context of health management positively influences an individual’s perceived usefulness of PHR systems.

H4: A higher level of BNS in the context of health management negatively influences an individual’s perceptions of complexity of PHR systems.

In summary, the proposed research model suggests that behavioral intention to start using a PHR system is influenced by an individual’s perceptions regarding usefulness (ie, PU) and effort (ie, CPLX) associated with using the system. Prior research on IS adoption suggests that these perceptions (ie, internal beliefs about the system or individual reactions to using the system) mediate the influence that external variables might have on behavioral intention [79,83,117]. Therefore, BNS is incorporated in the research model of Figure 2, as an external variable and an antecedent of PU and CPLX. Further, physician autonomy support and autonomous causality orientation are incorporated in the proposed research model in Figure 2 as determinants of BNS [45]. The model also includes self-efficacy as another external variable influencing PU and CPLX.

## Methods

### Research Design

In order to test the hypotheses in the proposed research model in Figure 2, this research employs a cross-sectional survey method. Surveys are the typical approach to empirically validate adoption models [118]. In addition, surveys are one of the most widely used methods in IS research [119]. Data collection was done through an online survey to gather measurement scales for the model factors as well as gather individual characteristics (eg, demographics, details of previous computer and Internet use) and control variables. Since the focus of this research is on understanding the “pre-usage” stage of PHR system adoption process, the online survey was administered to individuals with no prior experience in using any type of electronic PHR systems. Multimedia Appendix 2 presents the description of the online survey used in this study according to the Checklist for Reporting Results of Internet E-Surveys (CHERRIES) [120].

In order to reduce the effect of common method variance and to reduce the cognitive load on participants, the entire survey was divided into two parts, such that each part would be completed by participants in one sitting. For each participant, the two sittings were on average 36 hours apart. Each of the survey parts contained only approximately half of the questions. Using LimeSurvey, an open source survey app, the two parts of the survey for this study were programmed and were hosted on the McMaster University (Hamilton, Ontario, Canada) website. Finally, for the purpose of this study, PHR systems were introduced to participants using an online video clip that was shown to participants at the beginning of Part 2.

On entering the website for Part 1 and signing the consent form to participate in the study, participants were presented with a set of questions to determine their eligibility for this study. Only persons living in Canada (the target population of this study), above the age of 18 years (ethical consideration), with a family physician (the measurement items of physician autonomy support relate directly to the participants’ family physician), and with no prior experience in using electronic PHR systems of any type (our focus is on pre-usage stage of PHR system adoption) were considered eligible to participate in this study. Ineligible participants were prevented from starting the survey.

Since this study was targeted at individuals with no prior experience in using PHR systems (ie, this study was focused on a pre-usage stage of PHR adoption), an online video clip was created (described in Multimedia Appendix 2) and used to introduce such systems to study participants. The purpose of the video clip was to provide participants with introductory information about PHR systems and to show them how a PHR system can be used through a few real-life scenarios. The scenarios were developed in a way that covered the major functionalities of a typical PHR system as well as what operating a PHR account would entail (eg, keeping the account up to date). It is suggested that, in the absence of an actual system, video mockups can help shape the perceptions of consumers regarding the system [79]. Such video mockups can be used to “create realistic facades of what the system consists of.” Further, introducing PHR systems to study participants using a video clip was favored over using text-based material, still images, and slides. Multimedia material, such as video clips, can introduce the dynamic features of a product (eg, a PHR system) to consumers in a richer format [121]. Increasingly, commercial websites employ video clips to present product features since using a video clip provides greater vividness in presenting product features to consumers compared to text-based material and static images [122], and as a result, can help consumers understand and evaluate the quality and performance of products sold online [123,124].

### Instrument

In order to ensure content validity, measurement scales were selected from the existing literature, and in some cases, were slightly adapted to reflect the context of this study. The full measurement instrument can be found in Multimedia Appendix 2. In addition to the questions related to the proposed model, our survey also included questions related to several control variables (ie, age, gender, extent of daily Internet use, Internet experience in years, education level, perceived health status, chronic illness, frequency of doctor visit, years with family doctor, family health responsibility, prior use of paper-based health records, information privacy concerns, information security concerns, household income, and retirement status). Given our focus, which is examining the role of SDT factors in PHR system adoption, privacy and security concerns were not included in the research model in order to preserve the parsimony of the proposed model. However, since several studies have suggested consumers’ privacy and security concerns to be major barriers of PHR system adoption, questions related to these two variables were included in the survey in order to control for the effects they might have had on PHR system adoption.

### Recruitment

Participants were recruited through a commercial market research firm with a consumer panel that includes over 400,000 Canadians (the target population of this study). Invitations to take part in this study were balanced based on participant location, age, and gender, according to the 2011 Canadian Census Profile provided by Statistics Canada [125]. Participants were invited via email, which helped overcome physical limitations in reaching a wider audience across the target population, to enhance the representativeness of the sample. The representativeness of the sample was further enhanced by random sampling of the target population, thus improving the generalizability of our findings [126].

Prior to conducting data collection for the study, a pre-test of the instrument was conducted by inviting PhD students and three IS faculty members at McMaster University to complete the survey and provide their feedback on the instrument. Their feedback and responses to survey questions resulted in minor revisions to the questions as well as data collection procedures. On finalizing the online survey, a pilot was conducted through the same commercial market research firm with the purpose of diagnosing any possible flaws in the data collection procedures. As a result, 20 participants filled out the survey. The pilot study did not result in any changes in either data collection procedures or the measurement instrument. Therefore, the 20 data cases were included in the final dataset for this study. Finally, prior to conducting any sort of data collection, an ethics application was approved by the research ethics board of McMaster University.

There are two criteria that would impose minimum sample size requirements on this research [127]: minimum number of data cases (ie, participants) required for running Partial Least Squares (PLS) analyses and minimum number of cases required to achieve an acceptable statistical power in detecting a desired effect size for the relationships in the proposed model. In this study, the larger of the two was determined to be 139 (cases required to achieve a statistical power of at least 80 in order to detect medium effect sizes for a model with 3 predictors) [127-130].

The recruitment of participants and the administration of the survey ran from August 1-17, 2012. In order to obtain the 139 cases, and following the recruitment firm’s prior experience, a total of 6423 persons were invited, of whom 508 individuals completed Part 1, and 173 completed Part 2 as well. Thus, the response rate for Part 1 of the survey was 7.91%; for Part 2, it was 34.06%. Although the response rate fits within the acceptable range for this type of research [119], in survey research, sample representativeness is more important than response rate [131]. Stratified random sampling is an approach that increases sample representativeness [132].

In order to examine the possible existence of nonresponse bias, respondents were compared to two groups of nonrespondents (ie, those invitees who did not complete Part 1 and those who did not complete Part 2). The comparisons were conducted based on socioeconomic information as suggested by Sivo et al [119]. As such, the means of socioeconomic information for the abovementioned groups were compared using independent-samples *t* tests [133]. Results of the comparisons showed no significant difference between respondents and nonrespondents. Hence, it was concluded that nonresponse bias was not a concern for generalizing our findings.

Before conducting the main analyses of this study, this dataset was investigated for data anomalies (eg, participant’s gaming patterns), univariate outliers, multivariate outliers, and cases with missing data [133]. In total, this data screening resulted in the elimination of 14 cases from the dataset. The remaining 159 valid data cases were used in all subsequent analysis procedures detailed in this paper (N=159).

### Statistical Analysis

#### Research Model Evaluation

Structural equation modeling (SEM) was used to validate our proposed research model. SEM allows for the analysis and investigation of unobservable variables that are indirectly measured from observable variables [134,135]. In particular, SEM approach of PLS was used in this study. The choice of SEM approach depends on the objectives of the research being conducted [136]. Accordingly, PLS was chosen for this study for the following reasons. First, PLS gives optimum prediction accuracy because of its prediction orientation [137], and this characteristic of PLS is well suited to our overall objective, which is to understand what factors would explain consumers’ intention to use PHR systems. Such prediction is offered in PLS by determining the portion of the variance in the endogenous variable that is explained by exogenous variables. Second, in situations where the phenomenon being researched is relatively new or where the theoretical model is in the early stages of development, the PLS approach is more suitable [129]. Both PHR systems and PHR system adoption are relatively new phenomena. Furthermore, the proposed research model was developed and evaluated for this study for the first time. Third, as mentioned earlier, the construct of BNS was modeled and measured in this study as a second-order construct. PLS is a strong and flexible approach for evaluating models with higher order constructs [127,138-140].

We conducted and reported PLS analyses following a two-step approach as suggested by Chin [138]. In the first step, quality of the measurement model was assessed in terms of reliability and validity (measurement model evaluation). In the second step, quality of the structural model was assessed as explained in the Results section (structural model evaluation). Our PLS analyses were conducted using SmartPLS software (Version 2.0.M3) due to its ease of use as well as its capability of executing the range of procedures reported in this paper [141].

#### Measurement Model Evaluation

As mentioned earlier, the BNS construct was modeled and measured as a second-order factor. The procedures of measurement model evaluation for the second-order factor must be the same as those performed for the first-order factor [138,142]. As a result, this section of the paper is divided into two parts of first-order measurement model evaluation and second-order measurement model evaluation.

##### First-Order Measurement Model Evaluation

The measurement model evaluation (Multimedia Appendix 3) started with the assessments, and as a result, confirmation of individual item reliability and construct reliability. Next, the first-order measurement model was evaluated, and as a result, confirmed in terms of validity.

##### Second-Order Measurement Model Evaluation

The PLS modeling of the second-order factor (BNS) was done following Agarwal and Karahanna [142] (page 678, footnote 2) and Calvo-Mora et al [143]. Results of the evaluation of the second-order measurement model confirmed individual item reliability, construct reliability, and discriminant validity. Details are presented in Multimedia Appendix 3, which also provides the descriptive statistics for the model constructs.

#### Common Method Bias

The survey for this study was designed following the guidelines suggested by Podsakoff et al [144] in order to minimize the threat of common method bias. The potential presence of common method bias in our findings was assessed using the Harman’s one factor test [145] and the unmeasured latent marker construct technique [144,146] (Multimedia Appendix 3). Results of conducting these two tests were not suggestive of the presence of common method bias.

## Results

### Participant Characteristics

The characteristics of the study participants (N=159) are presented in Tables 2 and 3.

### Structural Model Evaluation

**Figure 4:**
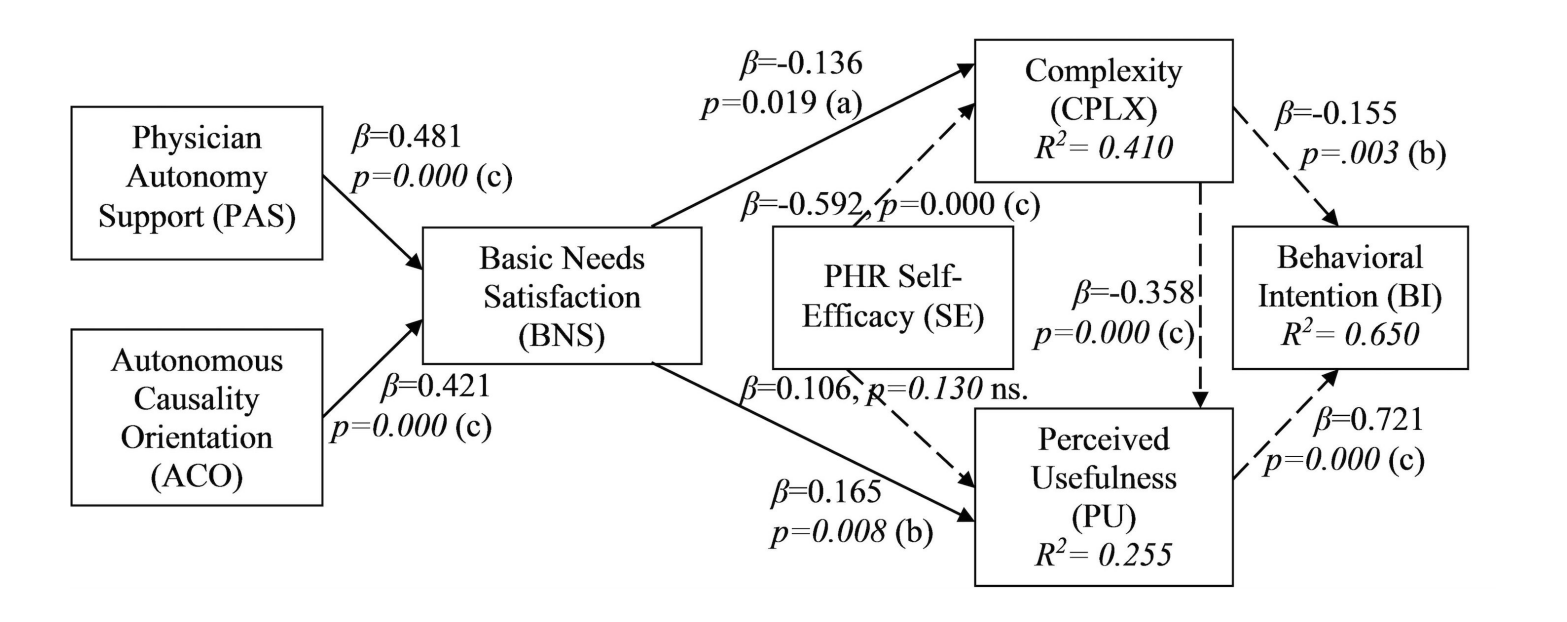
PLS results for the proposed research model: Significant at (a) .05; (b) .01; (c) .001 (ns=non-significant path).

Figure 4 presents the results of the PLS structural model evaluation of our proposed model. As shown, all the main hypotheses (H1-H4) are supported. The non-hypothesized relations from previous IS literature were also found to be significant with the exception of self-efficacy to PU.

Our research model was further examined, and as a result, confirmed in terms of predictive relevance, and goodness-of-fit (Multimedia Appendix 3).

Participants in this study were also asked questions about their individual characteristics as well as several control variables (ie, age, gender, extent of daily Internet use, Internet experience in years, education level, perceived health status, chronic illness, frequency of doctor visit, years with family doctor, family health responsibility, prior use of paper-based health records, information privacy concerns, information security concerns, household income, and retirement status). The impact of these individual and control variables on the results of this study was assessed by examining variations in the R^2^ for endogenous variables in the model or changes in the support for the hypothesized relations. Results of these examinations showed that the control variables and the individual characteristic variables did not change our findings. Results of control variable analysis are presented in Multimedia Appendix 3.

**Table 2:** Frequency statistics of participant characteristics.

Characteristics		Freq.	%	CC^a^	% Dev. from CC^b^
**Gender**					
	Female	83	52.20	51	2.35
	Male	76	47.80	49	2.44
**Age group**					
	18-34	48	30.2	27	11.85
	35-49	32	20.1	26	22.69
	50+	79	49.7	45	10.44
**Canadian province**					
	Alberta	18	11.3	10.5	7.61
	British Columbia	22	13.8	13.5	2.22
	Manitoba	6	3.8	3.5	8.57
	New Brunswick	2	1.3	2.5	48
	Newfoundland	1	0.6	1.5	60
	Nova Scotia	5	3.1	2.5	24
	Ontario	61	38.4	38.5	0.25
	Prince Edward Island	0	0	1	100
	Quebec	39	24.5	23.5	4.25
	Saskatchewan	5	3.1	3	3.33
**Education level^c^**					
	Secondary school or less	23	14.47		
	Some university or college	36	22.64		
	University or college degree	71	44.65		
	Some graduate work	4	2.52		
	Graduate degree	25	15.72		
**Annual household income (Can $)^c^**					
	Less than 40,000	35	22.01		
	40,000-79,999	68	42.77		
	80,000-119,999	36	22.64		
	120,000-159,999	17	10.69		
	˃160,000	3	1.89		

^a^CC: % in the 2011 Canadian census.

^b^Dev: deviation.

^c^Not included in sample stratification.

**Table 3:** Descriptive statistics of participant characteristics.

Characteristics	Min.	Max.	Mean	SD
Age in years	19	82	48.16	16.11
Internet experience in years	3	26	16.60	6.52
Time spent online hours per day	1	12	3.67	2.43

## Discussion

### Principal Results

Findings from this study are discussed here in two parts. First, the appropriateness of the research model of this study in terms of explaining PHR system adoption is discussed, followed by a discussion of the results of hypotheses.

#### Appropriateness of the Research Model

In terms of the appropriateness of our research model for explaining pre-usage adoption intentions, the overall R^2^ of the endogenous construct (behavioral intention) in the research model (.650) indicates that a large portion of the variance (65%) in this construct was explained by the factors in the model, thus indicating the high explanatory power of the research model. Further, the cross-validated redundancy for the endogenous variables in the research model (Q^2^), as well as the absolute and relative goodness-of-fit indices, is indicative of the model appropriately explaining an individual’s adoption of PHR systems.

#### Results of Hypotheses Testing

In terms of our hypotheses, consistent with prior research on IS adoption, PU and CPLX of PHR systems were shown to be the key antecedents of behavioral intention to use such systems. In addition, self-efficacy was shown to be a significant predictor of CPLX. In contrast, the association between self-efficacy and PU was not statistically significant. Compeau and Higgins [74] found a positive influence of self-efficacy on outcome expectations (conceptualized and measured similar to PU) where participants were recruited from individuals with various levels of experience in using the information system in question. The study was conducted on a pool of data not corresponding to a specific technology adoption stage, whereas our study focuses only on the pre-usage stage of adoption. Therefore, self-efficacy may not have a significant effect on PU in the pre-usage stage. To support this finding, it is worth mentioning that Venkatesh [80] has shown that the effect of self-efficacy on behavioral intention in pre-usage stage is fully captured by the expected effort associated with using the system. It can similarly be argued that the effect of self-efficacy on PU in the pre-usage stage is fully captured by CPLX. This statement was tested and confirmed in this study by running a PLS analysis in the absence of CPLX in the research model. The result showed a statistically significant positive relationship between self-efficacy and PU (beta coefficient=.321, *P*<.001), which is in support of the above argument.

As argued in the theoretical development section of this paper, CPLX was incorporated in our model instead of the commonly used PEOU construct as representative of effort associated with using a PHR system. However, PEOU data were also collected, and we ran our research model with PEOU as well. We found no difference between having either CPLX or PEOU. However, having CPLX in the model yielded stronger associations and higher explained variances compared to PEOU, which supports our theoretical arguments to use it instead of PEOU.

BNS was shown in this study to be significantly associated with PU (beta coefficient=.165, *P*<.01). These results suggest that individuals with higher levels of self-determination (associated with higher BNS) in their health management would find a PHR system more useful compared to those with lower levels of self-determination.

BNS was also shown to have a significant negative association with CPLX (beta coefficient=-.136, *P*<.05). These results suggest that individuals with higher levels of self-determination would perceive less effort in using a PHR system.

Physician autonomy support was shown to be a significant predictor of BNS in the context of health management (beta coefficient=.481, *P*<.001). Consistent with prior research driven by SDT in other contexts, the results suggest that individuals whose physicians are more supportive of their being more self-determined in managing their health would exhibit higher levels of BNS.

Finally, the personality trait of autonomous causality orientation was shown to be associated with BNS in the context of personal health management (beta coefficient=.421, *P*<.001). These results suggest that individuals with higher levels of autonomous causality orientation exhibit more self-determination in managing their health compared to those with lower levels.

### Contributions

From an academic perspective, this research contributes to the literature by developing and validating a research model that explains the adoption of PHR systems, from an SDT perspective. As such, the current study highlights the importance of considering the changing role of consumers from passive recipients of care to active partners in their own care when considering the adoption of PHRs. Although this model is specific to using PHR systems for managing one’s health, such a role change supported by information technology could be observed in contexts other than health care (eg, self-service technologies). Findings of this research highlight the importance of considering how information systems can facilitate the changing way people engage in certain behaviors when trying to understand the adoption of such systems. Accordingly, this study is the first to apply SDT in order to understand PHR system adoption.

Our findings also showed the importance of physician autonomy support in the adoption of PHR systems by individuals. Similarly, the importance of considering the role of personality traits of autonomous orientation in PHR system adoption was shown. Finally, the measurement scales for the constructs of SDT were adapted and validated for the context of health management and can be used in similar future studies.

This study provides valuable implications and contributions to practice in terms of the development, promotion, and facilitation of PHR systems use by consumers. The major findings in terms of the supported hypotheses, the academic value added of testing each hypothesis, and the practical implications of the findings are summarized as follows.

Perceived usefulness positively influences behavioral intention, complexity negatively influences behavioral intention, and self-efficacy negatively influences complexity. This study provided empirical support for a relationship not previously validated in the context of using PHR systems for health management. In addition, the study adapted and validated self-efficacy scales for PHR systems. As for practical results, we suggest considering features deemed useful by consumers in designing PHR systems (eg, monitoring and tracking features), promoting PHR systems (highlight those features in advertisements), and facilitating PHR system use (eg, provide incentive for health care providers to communicate with patients through PHR systems), designing PHR systems that are easy to use and maintain, training consumers in using PHR systems, providing technical support and facilitating usage, and providing technical features that would reduce the ongoing effort of keeping the system up to date (eg, automatic data population, smart data population, compatibility with external devices such as blood sugar readers).

BNS negatively influences complexity, BNS positively influences perceived usefulness, physician autonomy support positively influences BNS, and autonomous causality orientation is positively associated with BNS. This study adapted and validated SDT scales for the context of personal health management and provided empirical support for relationships not previously investigated as well as providing empirical support for relationships not previously validated in the context of personal health management. In terms of practice, the results suggest that health care providers must generally allow their patients to take part in their health management. That said, it must be noted that according to SDT, people with different personality orientations are motivated through different regulation mechanisms. For example, for individuals with a personality orientation toward being controlled (rather than being autonomous), rewards and punishments may promote a higher level of self-determination in health management [45], consequently facilitating higher adoption rates for PHR systems.

### Limitations

This research was carried out in a Canadian context; thus, findings from the research will not be immediately transferrable to other countries with different demographics, health care system characteristics, and cultures. For example, the role of culture is believed to be influential in research related to SDT (eg, [147]), information systems in general (eg, [148]), and technology adoption in particular (eg, [149]). Hence, further research would be required before transferring the findings of this study to other countries.

Data collection for this study was conducted by employing a cross-sectional survey design. Given that perceptions and intentions (CPLX, PU, and behavioral intention) regarding the use of PHR systems could change over time, collecting data at one point could pose a threat of temporal instability in the findings. Nevertheless, the focus of the study was on only one particular stage in the adoption process where individuals had no prior experience with using PHR systems (ie, pre-usage), and the selected method of data collection was deemed to be the best approach in this case.

### Future Research Directions

The current study was focused on the pre-usage stage of PHR system adoption process. In this stage, consumers may not have a full understanding of the nature of the change in their roles (from passive to active) when using an actual PHR system. Therefore, possible venues of future research may include the development and validation of a theoretical adoption model for later stages of the adoption process (ie, initial use, continued use). Using PHR systems might influence an individual’s level of BNS in health management [27]. Thus, another area for future research is to investigate such an influence. In other words, research is needed to understand the influence of PHR system usage on an individual’s self-determination in managing their health.

Finally, several other factors could impact PHR adoption such as trust, security, privacy, and social influence. Although influences of some of these variables were investigated through their use as control variables in this study, future studies should explore their role more formally.

## Appendix

Multimedia Appendix 1 Positioning of the study in relation to the literature.

Multimedia Appendix 2 Measurement instruments and data collection.

Multimedia Appendix 3 Statistical analyses.

## Abbreviations

BNS: basic needs satisfaction
CPLX: complexity
IS: information systems
PEOU: perceived ease of use
PHR: personal health record
PLS: partial least squares
PU: perceived usefulness
SDT: self-determination theory
SEM: structural equation modeling
TAM: technology acceptance model
