# Supplementary material for: Consumer Adoption of Personal Health Record Systems: A Self-Determination Theory Perspective
Source: J Med Internet Res. 2017 Jul 27;19(7):e270. doi: 10.2196/jmir.7721 (PMC5553007; doi:10.2196/jmir.7721)
Supplement: Multimedia Appendix 1 [file jmir_v19i7e270_app1.pdf]

Multimedia Appendix 1: Positioning of the study in relation to the literature

In order to demonstrate the novelty of our study, this Multimedia Appendix positions our study in relation to the literature, by providing an overview of the literature from three different perspectives: (i) information systems/technology adoption, (ii) health information systems/technology adoption, and (iii) PHR system adoption.

(i) Information systems/technology adoption: Technology adoption is arguably the most extensively explored area of research within the IS discipline, and it has resulted in the development of rich theories that explain individual technology adoption and use (e.g., [1-7]). Specifically, the technology acceptance model (TAM) [3] has been most widely employed as the basis to investigate individual IT adoption behavior [4]. TAM posits that behavioral intention to use a system (i.e., adoption) is mainly explained by internal user beliefs (namely perceived usefulness and perceived ease of use) regarding the use of the system. Further, external variables (e.g., system characteristics) influence adoption decisions mainly through influencing those internal variables [2]. Venkatesh and Bala [4] synthesized TAM-based studies in order to accumulate the body of knowledge regarding such external variables into a theoretical framework which resulted in suggesting four categories of external variables (i.e., determinants of perceived usefulness and perceived ease of use): individual differences, system characteristics, social influence, and facilitating conditions. We argue that "motivation to take a more active role" while engaging in a behavior (e.g. one's own health management) for which an information system (e.g. PHR) is designed to support does not fall within any of these categories. Thus, and following the discussion in the introduction of this paper, there is a need to extend current technology acceptance research to account for the role of the change in the user's motivation to take a more active role while engaging in the behaviour for which an IS is being used and the influence of such motivation and associated role change in motivating the adoption of the IS in question.

(ii) health information systems/technology adoption: Adoption of health information systems/technology has also been studied by several authors in recent years [8]. Or and Karsh [9] performed a systematic literature review in order to identify factors which would promote the acceptance of health information systems for patients/consumers. That literature review resulted in categorizing influential factors into the following four groups: patient factors (i.e., individual differences), organizational factors (health care provider organization), environmental factors (limited to the geographical proximity to health services), and task factors (changes in the nature of the task supported by technology). This last factor, which is the focus of our paper, was suggested by the aforementioned systematic review to influence patient/consumer beliefs regarding the technology. However, the authors of this systematic review article mention that they did not find any study that empirically examined the effect of such factors on health system adoption and, as also suggested by [10], they call for further research in this area. As discussed above, the current study is a step towards empirically examining this suggested potential effect.

(iii) PHR system adoption: Since the beginning of the 21st century, there has been a surge in research on electronic personal health records [11, 12]. Although there are some overlaps, existing studies devoted to PHR systems can be generally categorized into seven categories of “definitions and descriptions”, “architecture and technical issues, “vision statements and research agendas”, “value and effect (e.g., on health outcomes, health care processes)”, “adoption and attitudes”, “evaluation (e.g., evaluation of functionality)”, and “privacy and security issues”. This categorization is expanded and updated by the authors based on the categorizations offered in [13] and [12]. Existing PHR related studies that investigate consumers’ “adoption and attitudes” are mainly concerned with identifying specific factors that would either facilitate or hinder PHR system adoption. In contrast, our research is aimed at developing a theoretical model which would help to understand mechanisms, rather than factors, through which PHR systems’ adoption could be enhanced. Of the existing PHR adoption studies, the empirical ones with theoretical foundation are provided in Table 1 below. Table 1 helps position our research within the existing literature on this topic. Our review of the PHR adoption literature reveals that no studies have empirically examined that the role of the change in the users’ motivation to take a more active role in managing their health through the use of PHRs on their decision to adopt PHRs. As such, our study addresses this gap in the information systems and PHR adoption literatures.

Table 1: Positioning of this paper in relation with current theoretical studies on PHR system adoption

**Study**	Objective
	Theory(ies) Used
	Findings
	Context
	What We Add
**[14]**	
	To conduct an exploratory empirical study on the applicability of DOI to the study of perceptions of PHRs, and to assess whether perceptions of PHRs predict the perceived value of the PHR for communicating with the doctor’s office.
	Diffusion of Innovations (DOI) [15, 16]
	From DOI, Ease of Use, Relative Advantage, Trialability, and Observability were found to distinguish between users and non-users of PHR systems. Of these factors, the first three were found to be significant predictors of the value of a PHR for communicating with the doctor’s office
	A cross-sectional survey targeted at patients of several ambulatory care centres

	in United States who were offered tethered PHR service.
	Based on DOI and overall, this paper investigates whether personal innovativeness (willingness to try a new technology) matters in PHR adoption. However, the nature of the new technology is not considered. Our objective is to understand if the changing role of individuals in managing their own care (the nature of PHRs as new technology) matters in PHR adoption.
**[17]**	
	To explore the factors influencing adoption of USB-based PHRs in Taiwan.
	Technology Acceptance Model (TAM) [3], Theory of Planned Behavior (TPB) [18]
	From TAM and TPB, Perceived Usefulness, Subjective Norms, and Computer Self-Efficacy were found to be significant predictors of PHR usage intentions.
	A cross-sectional survey targeted at patients of outpatient departments at ten medical centers in Taiwan who were offered USB-based (standalone) PHR service.
	This study applies TAM and TPB to the context of adoption of PHRs. However, specific characteristics of PHR systems which would distinguish them from other type of IS are not considered. Our focus is on PHRs as an instance of an IS that are distinct in the sense that they would potentially change the role of its users (from passive to active) in managing their own health.
**[19]**	
	To examine the decision to use standalone PHR systems as a trust-enabled fair social contract.
	A trust-enabled fair social contract model
	The perceived benefits of using a standalone PHR, perceived privacy control and trust were found to be the major factors determining intention to adopt the PHR.
	A cross-sectional online survey targeted at students at a major northern U.S. university which asked participants about their perceptions regarding standalone PHR systems.
	This study examines the role of trust in PHR adoption, and as such, it looks at specific characteristics of PHR systems (containing sensitive health information) that distinguishes such systems from general IS. However, it still does not speak to the changing role of PHR users in their health management.
**[20]**	
	To explore which factors will influence the behavioral intentions of infertile

	patients to use the PHR systems.
	TAM
	Perceived Usefulness, Perceived Ease of Use, and Patient-Physician Relationship were found to influence (either directly or indirectly) intention to use PHR systems.
	A cross-sectional survey targeted at infertile patients of a medical care center in Australia.
	This article augments TAM with a single new factor (Patient-Physician Relationship, defined as "the extent of familiarity, trust, and interaction between physicians and patients in the context of healthcare planning"), and has shown a direct influence of this new factor on intention to use PHRs. In contrast, rather than what factor contributes to intention, we are focused on mechanisms trough which an individual would be motivated to use PHR systems.
**[21]**	
	To validate TAM for PHR systems.
	TAM
	Perceived usefulness and perceived ease of use were found to influence (either directly or indirectly) behavioral intention to use PHR systems.
	A cross-sectional survey regarding tethered PHR systems.
	This study applies TAM to the context of adoption of PHRs. In doing so, specific characteristics of PHR systems which would distinguish them from other types of information system is not considered. Our focus is on PHR as an instance of IS that would potentially change the role of its users (from passive to active) in managing their own health.
**[22]**	
	To propose and validate a theoretical model of PHR adoption.
	TAM, Unified Theory of Acceptance and Use of Technology (UTAUT) [23]
	Perceived usefulness, security, privacy, and trust in PHRs, together with personal information technology innovativeness, were found to be significant motivators of adoption, while computer anxiety was found to be a potential important deterrent.
	A cross-sectional survey targeted at patients afflicted with a chronic disease in Canada.
	The research model of this study is based in part on TAM and UTAUT, enhanced with technology constructs (personal IT innovativeness and Internet reliance), healthcare constructs (information seeking and satisfaction with medical care),

	and original constructs (access to data sources and security, privacy and trust). This study extends mainstream IS adoption models in order to identify which factors would influence PHR adoption. We are focused on mechanisms of motivating individuals to use PHR systems rather than identifying influential factors. In addition, our focus is on a change in the role of individuals (from passive to active) in their health management as a result of PHR use.

References

1. Venkatesh V Davis FD, A Theoretical Extension of the Technology Acceptance Model: Four Longitudinal Field Studies. Management Science; 2000, (46:2):186-204, doi:10.1287/mnsc.46.2.186.11926.
2. Davis FD, Bagozzi RPWarshaw PR, User Acceptance of Computer Technology: A Comparison of Two Theoretical Models. Management Science; 1989, (35:8):982-1003, doi:10.1287/mnsc.35.8.982.
3. Davis FD, Perceived Usefulness, Perceived Ease of Use, and User Acceptance of Information Technology. MIS Quarterly; 1989, (13:3):319-340.
4. Venkatesh V Bala H, Technology Acceptance Model 3 and a Research Agenda on Interventions. Decision sciences; 2008, (39:2):273-315.
5. Venkatesh V, Determinants of Perceived Ease of Use : Integrating Control , Intrinsic Motivation , and Emotion into the Technology Acceptance Model. Information Systems Research; 2000, (11:4):342-365.
6. Venkatesh V, Morris MG, Davis GBDavis FD, User acceptance of information technology: Toward a unified view. MIS Q; 2003, (27).
7. Brown SA, Dennis ARVenkatesh V, Predicting Collaboration Technology Use: Integrating Technology Adoption and Collaboration Research. Journal of Management Information Systems; 2010, (27:2):9-54.
8. Tavares J, and Oliveira, T., Electronic Health Record Patient Portal Adoption by Health Care Consumers: An Acceptance Model and Survey. Journal of medical Internet research; 2016, (18:3).
9. Or CK Karsh B-T, A systematic review of patient acceptance of consumer health information technology. Journal of the American Medical Informatics Association; 2009, (16:4):550-560.
10. Baird A, North FRaghu TS. Personal Health Records (PHR) and the Future of the Physician-Patient Relationship. in iConference. 2011. Seattle, WA, USA: ACM Press.
11. Tang PC, Ash JS, Bates DW, Overhage JMSands DZ, Personal Health Records: Definitions, Benefits, and Strategies for Overcoming Barriers to Adoption. Journal of the American Medical Informatics Association; 2006, (13:2):121-6, doi:10.1197/jamia.M2025.
12. Kim J, Jung HBates DW, History and Trends of "Personal Health Record" Research in PubMed. Healthcare Informatics Research; 2011, (17:1):3-17, doi:10.4258/hir.2011.17.1.3.

13. Kaelber DC, Jha AK, Johnston D, Middleton BBates DW, A Research Agenda for Personal Health Records (PHRs). Journal of the American Medical Informatics Association; 2008, (15:6):729-736, doi:10.1197/jamia.M2547.Introduction.
14. Emani S, Yamin CK, Peters E, Karson AS, Lipsitz SR, Wald JS, Williams DHBates DW, Patient Perceptions of a Personal Health Record: A Test of the Diffusion of Innovation Model. Journal of medical Internet research; 2012, (14:6).
15. Rogers EM, Diffusion of Innovations. 4th ed. 1995: The Free Press.
16. Moore GC Benbasat I, Development of an Instrument to Measure the Perceptions of Adopting an Information Technology Innovation. Information Systems Research; 1991, (2:3):192-222.
17. Jian W-S, Syed-Abdul S, Sood S, Lee P, Hsu M-H, Ho C-H, Li Y-CWen H-C, Factors Influencing Consumer Adoption of USB-based Personal Health Records in Taiwan. BMC Health Services Research; 2012, (12:1):277-277.
18. Ajzen I, The Theory of Planned Behavior. Organizational Behavior and Human Decision Processes; 1991, (50:2):179-211.
19. Li H, Gupta A, Zhang JSarathy R, Examining the Decision to Use Standalone Personal Health Record Systems as a Trust-enabled Fair Social Contract. Decision Support Systems; 2012, (In Press), doi:10.1016/j.dss.2012.10.043.
20. Liu C-F, Tsai Y-CJang F-L, Patients' acceptance towards a web-based personal health record system: An empirical study in Taiwan. International journal of environmental research and public health; 2013, (10:10):5191-5208.
21. Noblin AM, Wan TTFottler M, Intention to use a personal health record: a theoretical analysis using the technology acceptance model. International Journal of Healthcare Technology and Management; 2013, (14:1):73-89.
22. Archer N Cocosila M, Canadian Patient Perceptions of Electronic Personal Health Records: An Empirical Investigation. Communications of the Association for Information Systems; 2014, (34:1):20.
23. Venkatesh V, Morris MG, Davis GBDavis FD, User Acceptance of Information Technology: Toward a Unified View. MIS Quarterly; 2003, (27:3):425-478.
