# Supplementary material for: Consumer Adoption of Personal Health Record Systems: A Self-Determination Theory Perspective
Source: J Med Internet Res. 2017 Jul 27;19(7):e270. doi: 10.2196/jmir.7721 (PMC5553007; doi:10.2196/jmir.7721)
Supplement: Multimedia Appendix 2 [file jmir_v19i7e270_app2.pdf]

Multimedia Appendix 2: Measurement Instruments and Data Collection

Measurement Instrument

Data for this study was collected from three groups of variables: technology adoption variables, self-determination theory variables, and control variables. To ensure content validity, measurement instrument for these variables were adapted from the extant literature as detailed below.

Measurement scales for technology adoption variables

Behavioral Intention (BI), Source: [1]

BI1: If available to me, I intend to use an online PHR in the near future to help manage my health.

BI2: If available to me, I predict I would use an online PHR in the near future to help manage my health.

BI3: If available to me, I plan to use an online PHR in the near future to help manage my health.

Perceived Usefulness (PU), Source: [2]

PU1: Overall, I find an online PHR would be useful for managing my health.

PU2: I think an online PHR would be valuable to me in terms of managing my health.

PU3: The information contained in an online PHR would be useful for managing my health.

PU4: The functionalities provided by an online PHR would be useful for managing my health.

Complexity (CPLX), Source: [3]

CPLX1: Using an online PHR would take too much time from my normal duties.

CPLX2: Working with an online PHR seems so complicated; it would be difficult to understand what is going on.

CPLX3: Using an online PHR involves too much time doing mechanical operations (e.g., data input).

CPLX4: It would take too long to learn how to use an online PHR to make it worth the effort.

PHR Self-Efficacy (SE), Source: [4]

SE1: I am confident that I can use an online PHR if I was only provided with the online instructions for reference.

SE2: I am confident that I can use an online PHR even if there is no one around to show me how to do it.

SE3: I am confident that I can use an online PHR even if I have never used such a system before.

SE4: I am confident that I can use an online PHR if I have just seen someone using it before trying it myself.

SE5: I am confident that I can use an online PHR if I just have the online "help" function for assistance.

Measurement scales for self-determination theory variables

The original questionnaires from which we adapted our scales are protected by copyright. We were granted permission to use these scales for our study. However, we cannot publish the entire questionnaires. As a result, a few example items are provided here.

Basic Needs Satisfaction (BNS), Source: [5]

The scale includes 21 items of which 7 items relate to the need for autonomy, 6 items relate to the need for competence, and 8 items relate to the need for relatedness. As an example, below is an item that relates to the need for autonomy:
BNS_A1: I feel like I am free to decide for myself how to manage my health.

Physician Autonomy Support (PAS), Source: full-version [6], 6-item version [7]

The 6-item Health Care Climate Questionnaire (HCCQ) was used. Example item:
PAS_1: I feel that my physician provides me choices and options.

Autonomous Causality Orientation (ACO), Source: [8]

The three causality orientations (autonomy, control, and impersonal) were measured using the General Causality Orientations Scale (GCOS), 12, 7-point Likert items per causality orientation. For each of the three orientations, one score is calculated for each individual by summing the values of the corresponding 12 items.
Self-determination theory distinguishes between three types of causality orientations in each person: autonomous orientation, controlled orientation, and impersonal orientation. While the focus of this research is only on autonomous orientation, due to the design of the scale, questions related to all the three types must be asked together to ensure validity. Example item for autonomy:
ACO_1: Recently a position opened up at your place of work that could have meant a promotion for you. However, a person you work with was offered the job rather than you. In evaluating the situation, you're likely to think: You would probably take a look at factors in your own performance that led you to be passed over (very unlikely to very likely).

Measurement of control variables of this study

Perceived Health Status, Source: [9, 10]

1- How would you evaluate your health in general? (7-point “bad” to “excellent”)
2- Compared to women/men your age how would you evaluate your health? (7-point “bad” to “excellent”)

Chronic Illness, Source: [11, 12]

Do you currently live with any chronic condition/disease? (“Yes”, “No”, “Prefer not to say”)

Frequency of Doctor Visit, Source: [9]

How many times have you visited your family doctor in the past 12 months? (Never, Once, Twice, Three times, Four times, Five times, and More than five times)

Years with Family Doctor, Source: None (This question was included specifically to control the influence of it on the physician autonomy support variable).
How many years have you been with your current family doctor? (Number of years)

Family Health Responsibility, Source: None (This question was included to control for the influence of it on the perceived usefulness of integrated PHR systems).
Are you responsible for managing the health of anybody other than yourself? Examples: Parents, children, other family members. (Yes, No)

Use of Paper Records, Source: None (This question was included to control for the influence of a prior somewhat similar experience in managing health record.)
Do you currently collect or have you previously collected your health records in a paper-based form? (Yes, No)

Information Privacy Concerns, Source: [13-15]
For each statement below, select the option that best describes your opinion about an online PHR similar to the one in the video clip. (7-point “strongly disagree” to “strongly agree”)
1-I am concerned that I would have to store too much information about myself in an online PHR account.
2-I am bothered that I would have to store my personal information in an online PHR account.
3-I am concerned about my privacy when using an online PHR.
4-I have doubts as to how well my privacy would be protected on an online PHR.
5-My personal information could be misused if I use an online PHR.
6-My personal information could be accessed by unknown parties if I use an online PHR.

Information Security Concerns, Source: [13-15]
For each statement below, select the option that best describes your opinion about an online PHR similar to the one in the video clip. (7-point “strongly disagree” to “strongly agree”)
1-I would feel secure in providing sensitive information (e.g., my health records) when using an online PHR.
2-I would feel totally safe providing sensitive information about myself when using an online PHR.
3-I would feel secure sharing sensitive information on an online PHR.
4-The security issue of sensitive information would be a major obstacle to my using an online PHR.
5-Overall, an online PHR is a safe place to store/send sensitive information.

Given the focus of this study which is examining the role of self-determination theory factors in PHR system adoption, privacy and security concerns were not included in the research model of this study in order to preserve the parsimony of the proposed model. However, since several studies have suggested consumers’ privacy and security concerns to be major barriers of PHR system adoption, questions related to these two variables were included in the survey in order to control for the effects they might have had on integrated PHR system adoption.

Household Income, Source: [9]
What is your household income (Canadian Dollars)? (Less than $40,000, $40,000 - $79,999, $80,000 - $119,999, $120,000 - $159,999, More than $160,000, Prefer Not to Say)

Retirement, Source: [9]
Are you retired? (Yes, No, Prefer Not To Say)

PHR Introduction Video Clip

Since this study was targeted at individuals with no prior experience in using PHR systems, an online video clip was created and used to introduce such systems to study participants. The purpose of the video clip was to provide participants with introductory information about integrated PHR systems and to show them how an integrated PHR system can be used through a few real life scenarios. The video clip demonstrated the completion of the scenarios on an HTML prototype of a fictitious integrated PHR system. The video clip was displayed to the participants as part of completing the survey of this study.

Davis et al. [16] suggest that, in the absence of an actual system, video mockups can help shape the perceptions of consumers regarding the system. Such video mockups can be used to "create realistic facades of what the system consists of". Further, introducing integrated PHR systems to study participants using a video clip was favoured over using text-based material, still images, and slides. Multimedia material, such as video clips, can introduce the dynamic features of a product (e.g., an integrated PHR system) to consumers in a richer format [17]. Increasingly, commercial websites employ video clips to present product features [18, 19]. Using a video clip provides greater vividness in presenting product features to consumers compared to text-based material and static images [19], and as a result, can assist consumers in understanding and evaluating the quality and performance of products sold online Jiang and Benbasat [18], [20].

The developed video clip was further revised based on feedback received from a number of experts in order to ensure the contents of the clip represented typical functionalities of an online integrated PHR system. The experts included four information systems faculty members at the authors' university, with extensive research experience in the areas of IS adoption, eHealth, and PHR systems. In addition, three MSc e-Health students working as interns on an active open-source integrated PHR system, provided feedback on the video clip.

The followings are some technical considerations regarding the video clip. First, the final video clip was 13 minutes and 25 seconds long. Second, the video was uploaded onto the video sharing website YouTube, and it was embedded in one of the pages of the online survey. Third, a JavaScript code was embedded in the video clip that, for an amount of time equal to the length of the clip, prevented participants from moving on to the next page of the survey website. Fourth, all the video playback control buttons were disabled to ensure participants did not skip any part of the video while watching it. Fifth, the video dimensions were set to automatically fit to the screen size of the viewer (i.e., the maximum possible size for each viewer). Sixth, the quality and specifications of the video were tested on several different types of computers and

hand-held devices, with various screen sizes, screen resolutions, operating systems, and web browsers.

Checklist for Reporting Results of Internet E-Surveys (CHERRIES)

A checklist of recommendations for authors is presented by the Journal of Medical Internet Research (JMIR) in an effort to ensure complete descriptions of Web-based surveys. Papers on Web-based surveys reported according to the CHERRIES statement will give readers a better understanding of the sample (self-)selection and its possible differences from a "representative" sample. Below is the description of the online survey of this study according to the CHERRIES checklist [21].

Design

Design: Online, non-open survey targeted at the Canadian general public, administered by a commercial market research firm.

IRB Approval

IRB approval: Ethics approval was obtained at the authors' university.

Informed Consent: Participants were informed about all the details before signing up to fill out the survey.

Data protection: No sensitive data was collected, and all collected data were stored and controlled by the authors.

Development and testing

Development and testing: This was done over a number of rounds. Questions were checked by experts, and the procedures were designed by the commercial market research firm. The study was piloted twice: once with academic participants and once with participants recruited through the same market research firm.

Recruitment

Closed survey: Only those invited by the market research firm were presented with a link to participate in the study.

Contact mode: initial contact with potential participants was done by the market research firm, and it was through a number of ways including online, direct mail, TV advertisement, etc.

Advertising the survey: The link to participate in the survey was sent out to those enrolled in the market research firm's programs.

Survey administration

Web/E-mail: Survey was hosted online, and it was completed using designated web forms.

Context: The survey was hosted on the website of the authors' university.

Mandatory/voluntary: Participants were free to leave at any point.

Incentives: This was paid by the market research firm according to their agreement with participants.

Time/Date: August 2012.

Randomization of items or questionnaires: Questionnaires.

Adaptive questioning: No.

Number of Items: 75.

Number of screens: 12.

Completeness check: not done.

Review step: not available.

Response rates

Unique site visitor: 4337

View rate: 1

Participation rate: %42

Completion rate: %8

Preventing multiple entries from the same individual

Cookies used: by the recruiting firm.

IP check: done by the recruiting firm.

Log file analysis: done by the recruiting firm.

Registration: done by the recruiting firm.

Analysis

Handling of incomplete questionnaires: completing all questions was mandatory.
Questionnaires submitted with an atypical timestamp: one case was removed by the author for being very short (~2 minute for the entire questionnaire).

Statistical correction: none used.

References

1. Venkatesh V, Morris MG, Davis GBDavis FD, User acceptance of information technology: toward a unified view. MIS Quarterly; 2003, (27:3):425-478.
2. Pavlou PA, Consumer acceptance of electronic commerce: integrating trust and risk with the technology acceptance model. International Journal of Electronic Commerce; 2003, (7:3):101-134.
3. Thompson RL Higgins CA, Personal computing: toward a conceptual model of utilization. MIS Quarterly; 1991, (15:1):125-143.
4. Tan M Teo TSH, Factors influencing the adoption of internet banking. Journal of the AIS; 2000, (1:1es):5-5.
5. Deci EL, Ryan RM, Gagne M, Leone DR, Usunov JKornazheva BP, Need satisfaction, motivation, and well-being in the work organizations of a former eastern bloc country: a cross-cultural study of self-determination. Personality and Social Psychology Bulletin; 2001, (27:8):930-942, doi:10.1177/0146167201278002.
6. Williams GC, Grow VM, Freedman ZR, Ryan RM, Deci ELothers, Motivational predictors of weight loss and weight loss maintenance. Journal of Personality and Social Psychology; 1996, (70:1):115-126, doi:10.1037/0022-3514.70.1.115.
7. SDT Website, The health care climate questionnaire (HCCQ), 2014 9-12-2014]; Available from: http://www.selfdeterminationtheory.org/pas-health-care-climate/.
8. Deci E Ryan RM, The general causality orientations scale: self-determination in personality. Journal of Research in Personality; 1985, (19):109-134, doi:10.1016/0092-6566(85)90023-6.
9. Lafky DB Horan TA. Prospective personal health record use among different user groups: results of a multi-wave study. in 41st Hawaii International Conference on System Sciences. 2008.
10. Kaplan G Baron-Epel O, What lies behind the subjective evaluation of health status? Social Science & Medicine; 2003, (56:8):1669-1676.
11. Morton AA, Examining acceptance of an integrated personal health record (PHR), in Nursing. 2011, University of Maryland, Baltimore.
12. Smith AB, Odlum M, Sikka M, Bakken SKanter T, Patient perceptions of pre-implementation of personal health records (PHRs): a qualitative study of people living with HIV in New York City. Journal of HIV/AIDS & Social Services; 2012, (11:4):406-423, doi:10.1080/15381501.2012.735166.
13. Asim M, Petkovic M, Qu MWang C. An interoperable security framework for connected healthcare. in Consumer Communications and Networking Conference (CCNC), IEEE. 2011. Las Vegas, NV.
14. Shoniregun CA, Dube KMtenzi F, Introduction to e-healthcare information security, CA Shoniregun, K Dube, and F Mtenzi, Editors. 2010, Springer. p. 1-27.
15. Pavlou PA, Liang HXue Y, Understanding and mitigating uncertainty in online exchange relationships: a principal-agent perspective. MIS Quarterly; 2007, (31:1):105-136.
16. Davis FD, Bagozzi RPWarshaw PR, User acceptance of computer technology: a comparison of two theoretical models. Management Science; 1989, (35:8):982-1003, doi:10.1287/mnsc.35.8.982.

17. Raney AA, Arpan LM, Pashupati KBrill DA, At the movies, on the web: an investigation of the effects of entertaining and interactive web content on site and brand evaluation. J Interactive Marketing; 2003, (17:4):38-53, doi:10.1002/dir.10064.
18. Jiang Z Benbasat I, Research note: investigating the influence of the functional mechanisms of online product Presentations. Information Systems Research; 2007, (18:4):454-470.
19. Jiang ZJ Benbasat I, The effects of presentation formats and task complexity on online consumers' product understanding. MIS Quarterly; 2007, (31:3):475-475.
20. Jiang Z Benbasat I, Virtual product experience: effects of visual and functional control of products on perceived diagnosticity and flow in electronic shopping. Journal of Management Information Systems; 2005, (21:3):111-147.
21. Eysenbach G, Improving the quality of web surveys: the checklist for reporting results of internet e-surveys (CHERRIES). J Med Internet Res; 2004, (6:3):e34, doi:10.2196/jmir.6.3.e34.
