# Supplementary material for: Consumer Adoption of Personal Health Record Systems: A Self-Determination Theory Perspective
Source: J Med Internet Res. 2017 Jul 27;19(7):e270. doi: 10.2196/jmir.7721 (PMC5553007; doi:10.2196/jmir.7721)
Supplement: Multimedia Appendix 3 [file jmir_v19i7e270_app3.pdf]

Multimedia Appendix 3: Statistical Analyses

Validation of the Research Model

Effect Sizes, Predictive Relevance, and Goodness of Fit

The research model of this study was further examined by calculating the effect sizes for model paths, predictive relevance, and goodness of fit (GoF). Table 1 presents the effect sizes for total effects. Table 2 presents the cross validated redundancy (Q^2) for the endogenous variables in the research model of this study. Q^2 was used to examine the predictive relevance of the structural model. $Q^2>0$ implies the model has predictive relevance, whereas $Q^2<0$ represents a lack of predictive relevance [1]. GoF of the model refers to the overall (both measurement and structural levels) prediction performance of the model. The absolute GoF for the proposed model was 0.610 indicating a high fit of the model. The absolute GoF values of 0.36, 0.25, and 0.1 are considered high, medium, and low fit respectively [2, 3]. The relative GoF was calculated to be 0.921. A value above 0.9 speaks in favor of the fit of the model [4].

Table 1. Effect sizes for total effects (Rows: independent variables, Columns: dependent variables)

	**BI**	**PU**	**CPLX**	**BNS**
**PU**	.571			
**CPLX**	.206	.168		
**SE**	.110	.116	.361	
**BNS**	.038	.063	.040	
**ACO**	.015	.027	.020	.239
**PAS**	.009	.012	.009	.280

Table 2. Cross validated redundancy (Q^2) for dependent variables

	**Q^2**
**BI**	.615
**CPLX**	.292
**PU**	.221
**BNS**	.340

Measurement Model Evaluation

Table 3: Results of individual item reliability assessment for the first order model

Construct	Item	Item Loading	Corrected Item-total Correlation
**BI**	BI1	.981	.957
	BI2	.975	.943
	BI3	.979	.952
**PU**	PU1	.945	.902
	PU2	.955	.919
	PU3	.934	.883
	PU4	.953	.915
**CPLX**	CPLX1	.856	.759
	CPLX2	.867	.740
	CPLX3	.868	.780
	CPLX4	.887	.772
**SE**	SE1	.690	.538
	SE2	.885	.804
	SE3	.898	.817
	SE4	.773	.655
	SE5	.892	.807
**BNS-Autonomy**	BNS_A1	.670	.555
	BNS_A2R*	Item dropped	
	BNS_A3	.844	.643
	BNS_A4R	Item dropped	
	BNS_A5	.821	.541
	BNS_A6	.575	.506
	BNS_A7R	.589	.501
**BNS-Competence**	BNS_C1R	.686	.473
	BNS_C2	.686	.536
	BNS_C3	Item dropped	
	BNS_C4	.638	.441
	BNS_C5R	.678	.447
	BNS_C6R	.820	.64
**BNS-Relatedness**	BNS_R1	.908	.828
	BNS_R2	.881	.770
	BNS_R3R	Item dropped	
	BNS_R4	.585	.495
	BNS_R5	.895	.849
	BNS_R6R	Item dropped	
	BNS_R7R	.608	.433

	BNS_R8	.832	.735
**PAS**	PAS1	.89	.842
	PAS2	.914	.875
	PAS3	.896	.843
	PAS4	.891	.844
	PAS5	.944	.918
	PAS6	.934	.906
**ACO**	ACO	ACO is a personality index with 12 items. The score for this variable should be the sum of its 12 items [5]. Consequently, this variable should be estimated in PLS with equal weights (i.e., 1) for each of the 12 items. As a result, individual item reliability, AVE, and CR do not apply to this variable. Nevertheless, Cronbach's alpha was estimated to be .803 for the 12 items which is above the threshold of 0.7 indicating acceptable construct reliability. Reliability of this scale is established many times in the literature [6].	

* "R": item was negatively worded, and it was reverse coded for the analysis.
BI: Behavioral Intention; PU: Perceived Usefulness; CPLX: Complexity; SE: PHR Self-Efficacy; BNS: Basic Needs Satisfaction; BNS_A: Basic Needs Satisfaction (Autonomy); BNS_R: Basic Needs Satisfaction (Competence); BNS_R: Basic Needs Satisfaction (Relatedness); PAS: Physician Autonomy Support; ACO: Autonomous Causality Orientation.
Conducting individual item reliability tests resulted in eliminating five items that did not meet the acceptance criteria of corrected item-total correlations>0.4; and item loadings>0.5 [7].

Table 4: Results of construct reliability assessment

**Construct**	**AVE**	**Composite Reliability (CR)**	**Cronbach's Alpha**
**BI**	.957	.985	.977
**PU**	.896	.972	.961
**CPLX**	.756	.925	.893
**SE**	.692	.917	.885
**BNS-Autonomy**	.502	.831	.761
**BNS-Competence**	.501	.830	.743
**BNS-Relatedness**	.635	.910	.877
**ACO**	N/A	N/A	.803
**PAS**	.831	.967	.959

All the constructs in the study met the acceptance criteria of construct reliability (Average Variance Extracted (AVE)>0.5; Composite Reliability (CR)>0.7; Cronbach's Alpha>0.7) suggesting that reliability holds for all the variables in this study [8-10].

Table 5: Assessment of validity of the first order model (Matrix of loadings & cross-loadings for the 1st-order measurement model, all loadings sig. at 0.001)

	**BI**	**PU**	**CPLX**	**SE**	**BNS-A**	**BNS-C**	**BNS-R**	**ACO**	**PAS**
**BI1**	.981	.784	-.472	.352	.149	.214	.204	.186	.118
**BI2**	.975	.773	-.478	.314	.107	.219	.175	.191	.096
**BI3**	.979	.773	-.500	.367	.134	.231	.214	.198	.128
**PU1**	.780	.945	-.443	.342	.198	.228	.216	.261	.134
**PU2**	.791	.955	-.405	.290	.198	.276	.255	.274	.121
**PU3**	.699	.934	-.487	.405	.245	.295	.298	.345	.130
**PU4**	.736	.953	-.444	.372	.199	.237	.228	.244	.058
**CPLX1**	-.498	-.403	.855	-.438	-.115	-.257	-.162	-.212	-.112
**CPLX2**	-.365	-.350	.867	-.627	-.157	-.368	-.144	-.328	-.042
**CPLX3**	-.455	-.419	.867	-.450	-.193	-.299	-.249	-.281	-.170
**CPLX4**	-.408	-.460	.888	-.610	-.179	-.292	-.201	-.338	-.152
**SE1**	.349	.292	-.458	.690	.161	.171	.236	.273	.061
**SE2**	.239	.287	-.569	.885	.168	.243	.178	.302	-.025
**SE3**	.279	.287	-.551	.898	.147	.238	.157	.209	-.020
**SE4**	.211	.282	-.423	.773	.132	.237	.189	.196	-.022
**SE5**	.375	.387	-.584	.892	.131	.228	.166	.204	-.001
**BNS_A1**	.079	.095	-.023	.143	.670	.423	.336	.261	.270
**BNS_A3**	.098	.180	-.159	.135	.844	.501	.468	.483	.403
**BNS_A5**	.170	.239	-.168	.114	.821	.377	.491	.374	.510
**BNS_A6**	-.074	.038	-.010	.066	.575	.246	.189	.104	.150
**BNS_A7R***	.074	.133	-.204	.168	.589	.436	.458	.247	.288
**BNS_C1R**	.216	.198	-.413	.296	.229	.686	.223	.173	.200
**BNS_C2**	.192	.202	-.107	.116	.418	.686	.443	.291	.241
**BNS_C4**	.230	.284	-.149	.136	.444	.638	.472	.303	.284
**BNS_C5R**	.070	.111	-.223	.176	.469	.678	.412	.373	.294
**BNS_C6R**	.107	.176	-.311	.209	.438	.820	.399	.404	.245
**BNS_R1**	.147	.178	-.165	.191	.391	.480	.908	.461	.578
**BNS_R2**	.164	.225	-.258	.254	.450	.501	.881	.481	.566
**BNS_R4**	.200	.166	-.038	.059	.297	.236	.585	.168	.375
**BNS_R5**	.151	.229	-.124	.124	.377	.450	.895	.449	.562
**BNS_R7R**	.217	.252	-.225	.196	.386	.442	.608	.473	.392
**BNS_R8**	.126	.210	-.185	.188	.390	.460	.832	.511	.514
**ACO**	.196	.296	-.336	.284	.462	.443	.499	1.000	.311
**PAS1**	.144	.155	-.133	.025	.442	.304	.491	.298	.890
**PAS2**	.075	.048	-.107	-.035	.415	.271	.505	.269	.914
**PAS3**	.134	.138	-.149	.022	.500	.402	.518	.322	.896
**PAS4**	.094	.114	-.073	-.074	.472	.322	.490	.264	.891

**PAS5**	.106	.086	-.141	-.001	.489	.347	.505	.264	.944
**PAS6**	.082	.097	-.137	.045	.426	.302	.489	.277	.934

* “R” indicates that the item was negatively worded, and it was reverse coded.

To assess validity, first, a matrix of item loadings and cross-loadings was generated and used to examine discriminant validity. The loading of each item on its associated factor was compared to its cross-loadings (loadings on other factors). All items had higher loadings on their associated factors compared to cross-loadings (rows of the matrix). In addition, all factors loaded higher with their associated items compared to other factors (columns of the matrix).

Table 6: Assessment of validity of the first order model (Construct correlation matrix and discriminant validity assessment for the first-order measurement model)

	**BI**	**PU**	**CPLX**	**SE**	**BNS-A**	**BNS-C**	**BNS-R**	**ACO**	**PAS**
**BI**	.978								
**PU**	.794	.947							
**CPLX**	-.494	-.470	.870						
**SE**	.352	.371	-.627	.832					
**BNS-A**	.133	.222	-.186	.177	.709				
**BNS-C**	.226	.273	-.350	.269	.568	.707			
**BNS-R**	.202	.263	-.217	.219	.687	.550	.797		
**ACO**	.196	.296	-.336	.284	.462	.443	.549	1.000	
**PAS**	.117	.117	-.136	-.003	.503	.359	.674	.311	.912

BI: Behavioral Intention; PU: Perceived Usefulness; CPLX: Complexity; SE: PHR Self-Efficacy; BNS: Basic Needs Satisfaction; BNS_A: Basic Needs Satisfaction (Autonomy); BNS_R: Basic Needs Satisfaction (Competence); BNS_R: Basic Needs Satisfaction (Relatedness); PAS: Physician Autonomy Support; ACO: Autonomous Causality Orientation.

The square root of the AVE of each construct was compared with correlations of that construct with other constructs in the model. As shown in Table 6, all construct are more strongly correlated with their own measures than other constructs indicating confidence in the discriminant validity of the items and the factors of the first-order measurement model.

Table 7: Reliability assessment for the second order model (Results of individual item reliability assessment for the second-order model – Basic Needs Satisfaction)

**Construct**	**Item**	**Loading**	**Corrected Item-total Correlation**
**BNS**	BNS1	.871	.712

	BNS2	.805	.609
	BNS3	.893	.699

Table 8: Reliability assessment for the second order model (Construct reliability for the second-order measurement model – Basic Needs Satisfaction)

	AVE	Composite Reliability (CR)	Cronbach's Alpha
BI	.957	.985	.977
PU	.896	.972	.961
CPLX	.756	.925	.893
SE	.692	.917	.885
BNS	.734	.892	.819
ACO	1.000	N/A	.803
PAS	.831	.967	.959

BI: Behavioral Intention; PU: Perceived Usefulness; CPLX: Complexity; SE: PHR Self-Efficacy; BNS: Basic Needs Satisfaction; PAS: Physician Autonomy Support; ACO: Autonomous Causality Orientation

Table 9: Validity assessment for the second order model (Matrix of loadings and cross-loadings for the second-order measurement model – All loadings significant at 0.001)

**Items ↓**	**Constructs**						
	**BI**	**PU**	**CPLX**	**SE**	**BNS**	**ACO**	**PAS**
**BI1**	.981	.785	-.472	.352	.221	.186	.118
**BI2**	.975	.773	-.478	.314	.195	.191	.096
**BI3**	.979	.773	-.500	.367	.227	.198	.128
**PU1**	.780	.945	-.444	.342	.249	.261	.134
**PU2**	.791	.955	-.405	.290	.284	.274	.121
**PU3**	.699	.934	-.487	.405	.327	.345	.130
**PU4**	.736	.953	-.445	.372	.258	.244	.058
**CPLX1**	-.498	-.403	.856	-.438	-.205	-.212	-.112
**CPLX2**	-.365	-.350	.866	-.667	-.251	-.328	-.043
**CPLX3**	-.455	-.419	.868	-.450	-.287	-.281	-.170
**CPLX4**	-.408	-.460	.888	-.610	-.258	-.338	-.152
**SE1**	.349	.292	-.458	.690	.225	.273	.061
**SE2**	.239	.287	-.568	.885	.226	.302	-.025
**SE3**	.279	.287	-.550	.898	.207	.209	-.020
**SE4**	.211	.282	-.422	.773	.216	.196	-.022
**SE5**	.375	.387	-.583	.892	.202	.204	-.001
**BNS1**	.133	.222	-.186	.177	.871	.462	.504
**BNS2**	.226	.273	-.350	.269	.805	.443	.359
**BNS3**	.202	.263	-.217	.219	.893	.549	.674
**ACO**	.196	.296	-.336	.284	.570	1.000	.311

Items ↓	Constructs						
	BI	PU	CPLX	SE	BNS	ACO	PAS
**PAS1**	.144	.155	-.134	.025	.543	.298	.890
**PAS2**	.075	.048	-.108	-.035	.528	.269	.913
**PAS3**	.134	.138	-.149	.022	.614	.322	.897
**PAS4**	.094	.114	-.074	-.074	.550	.264	.891
**PAS5**	.106	.086	-.141	-.001	.581	.264	.944
**PAS6**	.082	.097	-.138	.045	.521	.277	.934

BI: Behavioral Intention; PU: Perceived Usefulness; CPLX: Complexity; SE: PHR Self-Efficacy; BNS: Basic Needs Satisfaction; PAS: Physician Autonomy Support; ACO: Autonomous Causality Orientation.

Table 10: Validity assessment for the second order model (Construct correlation matrix and discriminant validity assessment for the second-order measurement model)

	BI	PU	CPLX	SE	BNS	ACO	PAS
**BI**	.978						
**PU**	.794	.947					
**CPLX**	-.494	-.470	.870				
**SE**	.352	.371	-.627	.832			
**BNS**	.219	.295	-.288	.257	.857		
**ACO**	.196	.296	-.336	.284	.570	1.000	
**PAS**	.117	.117	-.137	-.003	.612	.311	.912

BI: Behavioral Intention; PU: Perceived Usefulness; CPLX: Complexity; SE: PHR Self-Efficacy; BNS: Basic Needs Satisfaction; PAS: Physician Autonomy Support; ACO: Autonomous Causality Orientation

Table 11: Descriptive Statistics for Model Constructs

Items	Mean	SD
**BNS_A1**	5.509	1.242
**BNS_A3**	5.472	1.152
**BNS_A5**	5.044	1.352
**BNS_A6**	4.818	1.550
**BNS_A7R***	5.642	1.375
**BNS_C1R**	5.440	1.403
**BNS_C2**	4.428	1.380
**BNS_C4**	4.610	1.445
**BNS_C5R**	5.038	1.373
**BNS_C6R**	5.459	1.413
**BNS_R1**	5.283	1.264
**BNS_R2**	5.421	1.219
**BNS_R4**	4.044	1.608
**BNS_R5**	5.189	1.269

Items	Mean	SD
**BNS_R7R**	6.132	1.091
**BNS_R8**	5.434	1.145
**ACO**	67.535	8.597
**BI_1**	4.918	1.518
**BI_2**	4.969	1.486
**BI_3**	4.874	1.542
**PU1**	5.421	1.214
**PU2**	5.365	1.285
**PU3**	5.535	1.101
**PU4**	5.428	1.161
**CPLX1**	3.642	1.628
**CPLX2**	2.799	1.409
**CPLX3**	3.610	1.587
**CPLX4**	2.887	1.449
**SE1**	5.390	1.267
**SE2**	5.352	1.186
**SE3**	5.409	1.197
**SE4**	5.308	1.263
**SE5**	5.509	1.190
**PAS1**	5.384	1.311
**PAS2**	5.465	1.330
**PAS3**	5.403	1.212
**PAS4**	5.340	1.475
**PAS5**	5.371	1.399
**PAS6**	5.252	1.359

* “R” indicates that the item was negatively worded, and it was reverse coded for the analysis.

Assessment of Common Methods Bias

In order to minimize common methods variance (CMV), the following actions were taken. First, the survey of this study was divided into two parts, and each part was completed by participants in a separate sitting. The time between completing the two parts of the survey for each participant ranged from 12 hours to 8 days, with an average of 36 hours in between completing the two parts of the survey. Such a temporal separation of measurement [11] reduces the possibility of participants’ responding to Part 2 questions based on what they remember from Part 1 questions, thus reducing the effect of consistency motif on the responses.

Consistency motif is suggested to be a source of CMV [11] [12]. Second, the survey questions were ordered such that the questions for the endogenous variables were presented to participants before the questions for the exogenous variables. Such counterbalancing of the order of questions is suggested to reduce the threat of CMV [11]. Third, participants of this study were informed that data collection for this study was being conducted anonymously.

Protecting respondent anonymity and reducing evaluation apprehension is another factor that is suggested to reduce CMV [11]. Fourth, the risk of CMV was believed to be lessened by the inclusion of a number of negatively worded items in the measurement instrument of this study [13].

Although all attempts were made to alleviate the threat of CMV in this study based on guidelines suggested by Podsakoff, MacKenzie [11], the influence of CMV on the results of the study was assessed [14] using Harman's one factor test [12] and an unmeasured latent marker construct technique [11, 15]. Results of conducting these two tests were not suggestive of the presence of CMB in this study. Details of the two tests are presented below.

Harman's One Factor Test

All 26 items in the research model of this study were entered in a factor analysis. The unrotated solution to the PCA suggested 5 factors with eigenvalue greater than 1. The first factor accounted for 35.360 percent of the variance and the 5 factors together accounted for 78.224 percent of the variance in data. The eigenvalue of the last factor was 1.155. Several items loaded on components other than the first extracted factor. As a result, it was concluded that the study items do not load on a single general factor (i). Next, a factor analysis with one factor was performed and it explained 35.360 percent of the variance, while the 5 factor solution explained 78.224 percent of the variance. Concisely, the one factor solution did not explain more than half of the variance in the data set items (ii).

Unmeasured Latent Marker Construct Technique

The second technique used in this study to assess the presence of CMB was the unmeasured latent marker construct technique [11]. Following Liang, Saraf [15], this technique was implemented in this study using Partial Least Squares (PLS). As such, a new factor was added to the PLS model of this study in order to capture method influence. The indicators of this new factor (i.e., common method factor) consisted of all the indicators of other variables in the research model of this study. In addition, the common method factor was linked to all other factors in the model. In order to investigate for the presence of CMB [16], PLS results must be reviewed as follows. While Chin et al. [17] acknowledge that running this technique using PLS is common in the IS literature, they also question the usefulness of it in detecting CMB. Nevertheless, it was decided to employ this technique as conducting an additional test would increase the likelihood of detecting CMB in the event that it was present in the data set of this study.

First, statistical significances of factor loadings of the common method factor must be examined. Second, for each indicator, the variance explained by its principal factor must be compared to the indicator's variance explained by the method factor. Following Liang et al. (2003), the squared loadings of principal constructs were interpreted as the variance explained caused by the principal constructs (R_1^2), whereas the squared values of the method factor loadings were interpreted as the variance explained by method (R_2^2). CMB is unlikely to be a serious problem if the method factor loadings are statistically insignificant, and the indicators' principal variances are substantially greater than their method variances [11, 15, 16].

Results of employing the technique are presented in Table 12. As seen in the table, none of the method factor loadings are statistically significant. In addition, the average variance explained by the principal factors is 0.814, while the average variance explained by the common method factor is 0.003. The ratio of 248:1 shows a very small magnitude of variance explained by the method compared to variance explained by the principal constructs. Consequently, CMB is unlikely to be a concern for this study.

Table 12: Results of conducting the unmeasured latent marker construct technique for the assessment of common methods bias

**Construct**	**Indicator**	**Principal Factor Loading**	R_1^2	**Method Factor Loading**	R_2^2
**BI**	BI1	.978***	.957	.003 n.s.	.000
	BI2	.989***	.977	-.038 n.s.	.001
	BI3	.953***	.907	.034 n.s.	.001
**PU**	PU1	.932***	.868	.015 n.s.	.000
	PU2	.992***	.984	-.047 n.s.	.002
	PU3	.865***	.748	.088 n.s.	.008
	PU4	.997***	.994	-.055 n.s.	.003
**CPLX**	CPLX1	.898***	.807	.048 n.s.	.002
	CPLX2	.888***	.788	-.003 n.s.	.000
	CPLX3	.872***	.760	-.078 n.s.	.006
	CPLX4	.823***	.678	.036 n.s.	.001
**SE**	SE1	.585***	.342	.141 n.s.	.020
	SE2	.921***	.849	-.051 n.s.	.003
	SE3	.940***	.884	-.062 n.s.	.004
	SE4	.828***	.686	-.068 n.s.	.005
	SE5	.848***	.718	.064 n.s.	.004
**BNS**	BNS1	.940***	.883	-.101 n.s.	.010
	BNS2	.780***	.608	.055 n.s.	.003
	BNS3	.851***	.724	.048 n.s.	.002
**PAS**	PAS1	.870***	.757	.047 n.s.	.002
	PAS2	.938***	.880	-.054 n.s.	.003
	PAS3	.867***	.753	.055 n.s.	.003
	PAS4	.907***	.822	-.036 n.s.	.001
	PAS5	.948***	.898	-.007 n.s.	.000
	PAS6	.938***	.880	-.004 n.s.	.000
**ACO**	ACO1	1.000***	1.000	.000 n.s.	.000
**Average**			.814		.003

*** p<.001; n.s. non-significant; Average ratio = 248:1; BI: Behavioral Intention; PU: Perceived Usefulness; CPLX: Complexity; SE: PHR Self-Efficacy; BNS: Basic Needs Satisfaction; PAS: Physician Autonomy Support; ACO: Autonomous Causality Orientation.

Assessment of possible effects of control variables

Two different procedures were conducted using PLS in order to analyze the responses to these questions regarding individual characteristics and control variables, as explained below. The first procedure was conducted to investigate the impact of these variables on the research model in terms of the effect size of each of the variables on R^2 of the endogenous constructs in the research model. To this end, for each individual characteristic/control variable, one controlled model was created by adding the variable with paths leading to all constructs in the model. Each effect size is calculated by comparing the R^2 of the endogenous constructs in the uncontrolled model and in the controlled model [18]. Table 13 presents the results of this analysis. The second procedure was conducted to examine the relationship between individual characteristics/control variables and all the factors in the research model of this study. To this end, in PLS, individual characteristics/control variables were linked to every factor in the model one at a time. Table 13 presents the results of the conduced PLS analyses.

After conducting the above analyses, all the variables (individual characteristics and control variables) with significant paths to any of the variables in the proposed model of this study were subject to further analysis in PLS as follows. Following Liang, Saraf [15], first, the variables were added to the proposed model one by one, and each time the significant links from Table 13 were established and PLS algorithm was run. In no case were the results (significances) of the hypotheses of this study changed. Second, instead of adding the variables one by one, all the variables were added to the research model at once having established all the significant paths from Table 13. Similarly, running the PLS algorithm did not result in any changes to the results of the hypotheses of this study.

Table 13: Effect of control variables on R^2 of dependent variables (f^2)

	**Variable**	**BI**	**PU**	**CPLX**	**BNS**
**Individual Char.**	Age	.000	.003	.000	.000
	Gender (1=Female, 2=Male)	.012	.003	.000	.002
	Internet Usage Hours per Day	.003	.007	.005	.002
	Internet Experience in Years	.000	**.054**	.000	.011
**Control Variables**	Education Level	.000	.019	.012	.002
	Perceived Health Status	.000	.007	.017	**.092**
	Chronic Illness (1=Y, 2=N)	.000	.001	**.021**	.004
	Frequency of Doctor Visit	.014	.001	.000	.018
	Years with Doctor	.000	.001	.000	.000
	Family Health Responsibility (1=Y, 2=N)	.000	**.022**	.003	.000
	Use of Paper Records (1=Y, 2=N)	.009	.014	.014	.002
	Information Privacy Concerns	.012	**.063**	**.164**	.002
	Information Security Concerns	**.036**	**.122**	**.054**	.011
	Household Income	.000	.003	.014	.000
	Retired (1=Y, 2=N)	.003	.005	.000	.011

Bold values indicate considerable effects (f2>.02).

BI: Behavioral Intention; PU: Perceived Usefulness; CPLX: Complexity; SE: PHR Self-Efficacy; BNS: Basic Needs Satisfaction.

Table 14: Impact of control variables on model constructs

	Variable	Stat.	BI	PU	CPLX	SE	BNS	PAS	ACO
Individual Characteristics	Age	β	-.009	-.044	.003	-.138	.021	**.331**	**.288**
		p<	n.s.	n.s.	n.s.	n.s.	n.s.	.001	.001
	Gender (1=Female, 2=Male)	β	.067	-.039	-.008	.133	-.047	**-.160**	**-.176**
		p<	n.s.	n.s.	n.s.	.050	n.s.	.050	.050
	Internet Use: Hours per Day	β	.040	.070	.061	.133	.041	-.107	-.125
		p<	n.s.	n.s.	n.s.	n.s.	n.s.	n.s.	n.s.
	Internet Experience in Years	β	.020	**-.196**	.022	-.031	.080	.080	.032
		p<	n.s.	.010	n.s.	n.s.	n.s.	n.s.	n.s.
	Education Level	β	-.029	.116	.096	.030	.044	-.071	**.175**
		p<	n.s.	n.s.	n.s.	n.s.	n.s.	n.s.	.010
Control Variables	Perceived Health Status	β	-.012	-.076	.111	**.306**	**.215**	-.045	**.196**
		p<	n.s.	n.s.	n.s.	.001	.001	n.s.	.010
	Chronic Illness (1=Y, 2=N)	β	-.020	.016	.115	.111	.048	-.015	-.120
		p<	n.s.	n.s.	n.s.	n.s.	n.s.	n.s.	n.s.
	Frequency of Doctor Visit	β	.070	.029	-.018	-.067	**-.106**	.123	**.176**
		p<	. n.s.	n.s.	n.s.	n.s.	.050	n.s.	.050
	Years with Family Doctor	β	.013	-.031	-.018	-.024	-.003	**.171**	.021
		p<	n.s.	n.s.	n.s.	n.s.	n.s.	.010	n.s.
	Family Health Responsibility (1=Y, 2=N)	β	-.026	**-.126**	.048	-.062	.024	-.044	.033
		p<	n.s.	.050	n.s.	n.s.	n.s.	n.s.	n.s.
	Use of Paper Records (1=Y, 2=N)	β	-.054	-.101	.092	-.026	.026	-.060	-.117
		p<	n.s.	n.s.	n.s.	n.s.	n.s.	n.s.	n.s.
	Information Privacy Concerns	β	-.076	**-.236**	**.307**	**-.301**	-.036	-.136	-.100
		p<	n.s.	.010	.001	.001	n.s.	n.s.	n.s.
	Information Security Concerns	β	-.122	**-.313**	**.191**	**-.339**	-.079	**-.158**	-.043
		p<	n.s.	.001	.010	.001	n.s.	.050	n.s.
	Household Income	β	.026	.036	-.088	.042	.021	-.065	.015
		p<	n.s.	n.s.	n.s.	n.s.	n.s.	n.s.	n.s.
	Retired (1=Y, 2=N)	β	-.044	.064	-.014	**.151**	-.075	**-.178**	**-.175**
		p<	n.s.	n.s.	n.s.	.050	n.s.	.010	.010

β: PLS path coefficient; p: p-value; n.s.: non-significant; Bold values indicate significant relationships;

References

1. Chin WW, How to wite up and report PLS analyses, V Esposito Vinzi, et al., Editors. 2010, Springer Berlin Heidelberg: Berlin, Heidelberg. p. 655-690.
2. Tenenhaus M, Vinzi VE, Chatelin Y-MLauro C, PLS path modeling. Computational Statistics & Data Analysis; 2005, (48:1):159-205, doi:10.1016/j.csda.2004.03.005.
3. Wetzels M, Odekerken-Schroder GVan Oppen C, Using PLS path modeling for assessing hierarchical construct models: guidelines and empirical illustration. MIS Quarterly; 2009, (33:1):177-177.
4. Vinzi VE, Trinchera LAmato S, PLS path modeling: from foundations to recent developments and open Issues for model assessment and improvement, V Esposito Vinzi, et al., Editors. 2010, Springer Berlin Heidelberg: Berlin, Heidelberg. p. 47-82.
5. Deci E Ryan RM, The general causality orientations scale: self-determination in personality. Journal of Research in Personality; 1985, (19):109-134, doi:10.1016/0092-6566(85)90023-6.
6. Deci EL Ryan RM, The "what" and "why" of goal pursuits: human needs and the self-determination of behavior. Psychological Inquiry; 2000, (11:4):227-268, doi:10.1207/S15327965PLI1104_01.
7. Gefen D, Straub DWBoudreau M-C, Structural equation modeling and regression: guidelines for research and practice. Communications of AIS; 2000, (4:August):Article 7, pp. 1-78.
8. Nunnally JC Bernstein IH, Psychometric theory. 1994, New York, NY: McGraw-Hill.
9. Werts CE, Linn RLJöreskog KG, Intraclass reliability estimates: testing structural assumptions. Educational and Psychological Measurement; 1974, (34:1):25-33, doi:10.1177/001316447403400104.
10. Fornell C Larcker DF, Evaluating structural equation models with unobservable variables and measurement error. Journal of Marketing Research; 1981, (18:1):39-50, doi:10.2307/3151312.
11. Podsakoff PM, MacKenzie SB, Lee J-YPodsakoff NP, Common method biases in behavioral research: a critical review of the literature and recommended remedies. Journal of Applied Psychology; 2003, (88:5):879-903, doi:10.1037/0021-9010.88.5.879.
12. Podsakoff PM Organ DW, Self-reports in organizational research: rroblems and prospects. Journal of Management; 1986, (12:4):531-544, doi:10.1177/014920638601200408.
13. Lindell MK Whitney DJ, Accounting for common method variance in cross-sectional research designs. Journal of Applied Psychology; 2001, (86:1):114-121, doi:10.1037/0021-9010.86.1.114.
14. Straub DW, Creating blue oceans of thought via highly citable articles. MIS quarterly; 2009, (33:4):2-2.

15. Liang H, Saraf N, Hu QXue Y, Assimilation of enterprise systems: the effect of institutional pressures and the mediating role of top management. MIS Quarterly; 2007, (31:1):59-87.
16. Williams LJ, Edwards JRVandenberg RJ, Recent advances in causal modeling methods for organizational and management research. Journal of Management; 2003, (29:6):903-936, doi:10.1016/S0149-2063_03_00084-9.
17. Chin WW, Thatcher JBWright RT, Assessing common method bias: problems with the ULMC technique. MIS Quarterly; 2012, (36:3):1003-A11.
18. Chin WW, The partial least squares approach for structural equation modeling, GA Marcoulides, Editor. 1998, Mahwah, NJ: Lawrence Erlbaum Associates. p. 295-336.
